# Oral-gut microbiome dysbiosis in obese smokers reveals compartment-specific shifts

**DOI:** 10.1186/s13568-026-02048-y

**Published:** 2026-04-13

**Authors:** Mohammed Ramadan, Esraa K. Hassan, Salah Abdalla, Ali A. Abdelrhman Ahmed, Marwa Azab, Kareem A. Ibrahim, Ibrahim A. Amin, Mohamed Ebid Ali, Ahmed Eid Alharbi, Mohammed Salah

**Affiliations:** 1 Department of Microbiology and Immunology, Faculty of Pharmacy, Al-Azhar University, Assiut, Egypt; 2 Department of Microbiology and Immunology, Faculty of Pharmacy, Egyptian Russian University, Badr City, Egypt; 3 Department of Microbiology and Immunology, Faculty of Pharmacy, Suez Canal University, Ismailia, Egypt; 4 Department of Medical Laboratories Technology, College of Applied Medical Sciences in Yanbu, Taibah University, Medina, Saudi Arabia; 5 Department of Microbiology and Immunology, Faculty of Pharmacy, Port- Said University, Port-Said, Egypt

**Keywords:** Obesity, Smoking, Orointestinal axis, Dysbiosis, Oral microbiome, Gut microbiome

## Abstract

Obesity and smoking are major global health threats with synergistic impacts on metabolic disease. The combined effects of these factors on the oral-gut (Orointestinal) microbiome axis, a crucial interface for systemic immunity and metabolism, remain poorly characterized. In this cross-sectional study, 108 age- and sex-matched participants were stratified into four groups (*n* = 27/group): Non-Obese Non-Smokers (Control), Obese Non-Smokers, Non-Obese Smokers, and Obese-Smokers. Orointestinal microbiomes were profiled via 16 S rRNA sequencing of stool and oral rinse samples, followed by comprehensive bioinformatics analysis. Compartment-specific patterns existed in relation to smoking and obesity. The oral microbiome of Obese-Smokers exhibited a significant, dose-dependent reduction in diversity (34% loss at > 20 cigarettes/day; *padj =* 0.008), synergistic depletion of oral commensals such as *Elizabethkingia* (log_2_FC = − 6.33, *padj* = 0.0032) and enrichment of pathobionts such as *Escherichia_Shigella* (log_2_FC = 3.2, *padj =* 0.00068). In contrast, the gut microbiome maintained stable bacterial diversity across groups, despite profound compositional shifts (PERMANOVA, *padj* = 0.003), with smokers showing a significantly elevated Firmicutes/Bacteroidetes ratio (1.78 vs. 0.91 in controls; *padj* = 0.006). Critically, Obese-Smokers revealed a significant bidirectional pattern of the Orointestinal axis, specifically in the oral cavity, as evidenced by the significant Orointestinal co-occurrence of the oral pathobionts *Neisseria* and *Leptotrichia* (*r =* 0.67, *p =* 0.0001), which potentially highlights microbial covariation across habitats. A random forest model identified the oral taxon *F0058* (Saccharimonadaceae) as a potential biomarker for Obese-Smoker status (AUC = 0.975, *padj* = 0.017). Obesity and smoking are associated with synergistic orointestinal dysbiosis, pathobiont expansion, commensal loss, and potential microbial covariation across habitats. The vulnerable oral microbiome, specifically the *F0058* biomarker, offers a promising target for noninvasive risk stratification and intervention.

**Supplementary Information:**

The online version contains supplementary material available at 10.1186/s13568-026-02048-y.

## Introduction

The human microbiome defines the host’s metabolic characteristics and plays an important role in maintaining the host’s immune system’s homeostasis, and helps to resist infections at distinct body sites. Studying microbiota variations may help us understand how lifestyle and the environment impact microbial communities in healthy and sick people (Huang and Shi 2019; Ogunrinola et al. 2020; Shabayek et al. 2022). Among lifestyle factors, obesity and smoking represent two of the most significant global health threats, collectively contributing to millions of deaths annually. However, their combined impact on the human microbiome, particularly along the interconnected oral-gut axis, remains poorly characterized (Batatinha et al. 2019; Wang et al. 2021; Wu et al. 2016).

The two largest microbial ecosystems in the human body are the oral and gut microbiomes. According to the Human Microbiome Project (HMP), more than half of the bacteria in the human body live in the gastrointestinal (GI) tract (29%) or the oral cavity (26%) (Park et al. 2021). The gut microbiome, which is complicated and difficult to study, is critical to human health, as any changes in the composition and functionality of the gut microbiome impair colonization resistance and have been linked to several GI and non-GI disorders (Fan and Pedersen 2021; Hills et al. 2019; Seekatz et al. 2022). Furthermore, the oral cavity is one of the most critical interfaces between the human body and its surroundings, with diverse microbial compositions that are affected by various factors and provide insight into immunity and metabolism (Arweiler and Netuschil 2016; Neri-Rosario et al. 2023; Peng et al. 2022). In addition to serving as the start of digestion, the oral microbiome is important for supporting both systemic and oral health (Deo and Deshmukh 2019). Previously, investigations of the microbiome were limited to culture-dependent approaches, but the extensive bacteria present in the oral cavity could not be cultured via traditional cultivation methods, and the most significant features of the gut microbiome are not well understood owing to a lack of scientific tools for identifying noncultivable microbes; however, advances in new genomic technologies such as bioinformatics and next-generation sequencing have unleashed many aspects to better understand this discipline (Deo and Deshmukh 2019; Sreevatshan et al. 2022; Tawfik et al. 2023).

Obesity is a systemic disease that affects the entire body and is caused by an imbalance between energy intake and expenditure (Mohamed Lotfy et al. 2020; Muluke et al. 2016; Saad et al. 2021). Obesity was associated with significant changes in the oral and gut microbiomes. Furthermore, the inclusion of salivary samples, the gut microbiome, and consideration of the microbial community structure may increase our understanding of the mechanisms linking the microbiota to obesity and the influence of the gut microbiome on nutritional status (Bombin et al. 2022; Zsálig et al. 2023). Smoking is a major public health issue that currently exists throughout the world, where such a preventable cause of premature death impacts almost every organ system in the body (Al-Zyoud et al. 2020; Al Bataineh et al. 2020; Shaheen et al. 2023). The oral cavity is one of the first areas of the body to be exposed to cigarette smoke, making it particularly vulnerable to increased carcinogenesis, reduced mucosal immunity, and changes in the oral microbiome (Al Bataineh et al. 2020). Cigarette smoke is a complex chemical mixture that includes nicotine, aldehydes, polycyclic aromatic hydrocarbons, nitrosamines and heavy metals, which are inhaled into the lungs, causing several consequences, such as a reduction in endogenous antioxidants, an increase in lipid peroxidation, oxidative stress, and proinflammatory factors (Mohamed and El-Hamd Mohamed 2021). In addition, the ingestion of cigarette smoke into the GI tract induces dysbiosis via different mechanisms (Leite et al. 2022; Wu et al. 2016). Compared with nonsmokers, exposure to cigarette smoke has been shown to significantly affect the gut microbiome composition, as evidenced by the unique fecal microbiome compositions of smokers (Shapiro et al. 2022; Sublette et al. 2020; Wang et al. 2021). Additionally, cigarette smoke can disrupt the oral microbiome through interference with bacterial adherence to mucosal surfaces, increasing the acidity of saliva, depleting oxygen, promoting antibiotic resistance and resistance to host immune cells, where such dysbiosis has been linked to dental caries, periodontitis and systemic disorders in the lung, GI, and cardiovascular systems (Huang and Shi 2019; Shin et al. 2015; Wirth et al. 2020). Despite this knowledge, the combined effects of obesity and smoking on the orointestinal axis have not been systematically investigated using a well-controlled comparative design.

The human hand microbiota profile has been correlated with the oral and gut microbiome patterns, indicating that the hand serves as a route for fecal-to-oral microbial transmission (Park et al. 2021; Shaffer and Lozupone 2018). This bidirectional interaction has the power to shape or reshape the microbial ecosystem of both habitats, so we use ‘oral–gut axis’ to describe bidirectional oral–fecal associations. Both smoking and obesity are known to influence the composition and function of the human microbiome, particularly along the orointestinal axis, which includes both oral and gut microbial communities. The combined presence of these factors may contribute to more complex alterations in microbial ecology. Unlike many previous studies that investigated disease-associated changes in the intestinal microbiota alone, the present study aimed to assess the impact of smoking and obesity, individually and in combination, on the orointestinal axis microbiome using 16S rRNA sequencing of both oral rinse and stool samples. Specifically, we examine four distinct groups, namely, Obese-Smokers, Smokers, Obese individuals, and Controls, with a focus on characterizing the relationships between the oral and fecal microbiomes across these populations.

## Methods

### Ethics statement

All research protocols involving human subjects were reviewed and approved by the Research Ethics Committee at the Faculty of Pharmacy, Suez Canal University, Egypt (reference number: 202106MH1), and carried out in accordance with the Declaration of Helsinki’s tenets. Written informed consent was obtained from all participants.

### Subjects and recruitment

**Fig. 1:**
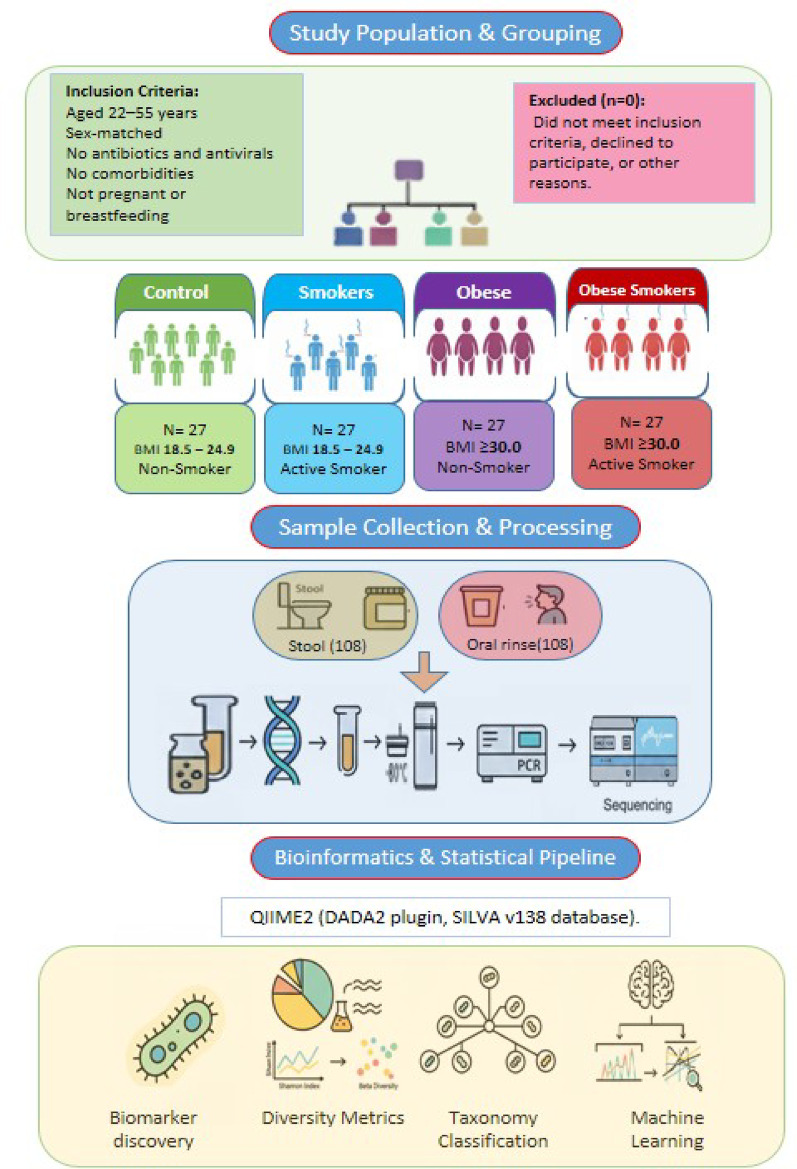
Schematic overview of study design and analytical workflow

This cross-sectional study recruited participants from Outpatient Clinics at Al-Salam District Medical Center in Ismailia, Egypt, and Badr University Hospital in Badr City, Egypt. Eligible subjects were aged 22*–*55 years (Fig. 1). Smokers were defined as individuals who smoked at least one cigarette daily and had a cumulative lifetime consumption of ≥ 100 cigarettes (Al-Zyoud et al. 2020; Qiu et al. 2022; Suzuki et al. 2022). Obesity was defined as a BMI of 31–45 kg/m^2^ (Aoun et al. 2020). The control subjects were nonsmokers (no tobacco use within the preceding 12 months) with a normal BMI (18.5*–*24.9 kg/m^2^, as per WHO criteria) and were group-matched where appropriate (Mohammed et al. 2022; Vasana et al. 2018).

To minimize potential confounding by diet, all participants completed the Stance4Health semi-quantitative food frequency questionnaire (FFQ), a self-administered tool specifically developed and validated to assess habitual dietary intake with a focus on gut microbiota (Formisano et al. 2024). A trained nutritionist also performed a 24-hour dietary recall to estimate daily energy and macronutrient intake (protein, fat, carbohydrate). Individuals reporting extreme dietary patterns (vegan or ketogenic) or significant changes in diet within three months before the study were excluded (Foster and Bradley 2018). Furthermore, statistical models were adjusted for total daily caloric intake to account for residual variation.

A power analysis, which was based on previous microbiome studies in obesity and smoking cohorts, indicated that a sample size of 25*–*40 participants per group would provide 80% power to detect moderate effect sizes (Cohen’s d ≥ 0.5) in alpha diversity metrics at a significance level of α = 0.05 (Kelly et al. 2015).

The study excluded subjects who met any of the following criteria: 1- medication usage within the past three months, including antibiotics, probiotics, corticosteroids and cytotoxic drugs; 2- a history of cancer, AIDS, hepatitis B or C viral infection, diabetes, hypertension, gastric surgery, pulmonary or cardiovascular diseases; and 3- the existence of dental caries, oral abscesses, untreated cavitated carious lesions, periodontal disease and periodontal pockets ≥ 4 mm (Al-Zyoud et al. 2020; Tawfik et al. 2023; Zhou et al. 2017).

### Sample collection

Stool samples were freshly collected in sterile 100 mL plastic containers, and oral rinse samples were obtained by instructing participants to swish and gargle with 10 mL of sterile 0.9% saline for 1 min. Oral rinse sampling was selected to capture an integrated view of the oral microbiota across multiple niches, facilitating direct comparison with gut luminal communities. Notably, our strict exclusion criteria, including the absence of periodontal pockets ≥ 4 mm, active caries, or oral abscesses, ensured that participants were periodontally healthy at baseline. This minimizes the risk that observed microbial alterations reflect localized oral disease rather than systemic exposures to smoking and obesity. While oral rinses may underrepresent adherent subgingival biofilms compared to targeted plaque sampling, this limitation is less critical in a periodontally healthy cohort. Furthermore, any underrepresentation would bias toward the null, making the significant dysbiosis we detected in smokers and Obese-Smokers all the more compelling. Future studies combining oral rinse with site-specific plaque sampling in diseased populations would provide complementary insights. The samples were chilled in an ice bag or tank during transport to maintain stability and minimize microbial shifts. Upon arrival, the chilled samples were immediately centrifuged at 10,000 × g for 10 min at 4 °C (Lu et al. 2022). The oral rinse samples were then transferred aseptically to sterile Eppendorf tubes, and finally, both the stool and oral rinse samples were stored at − 80 °C until the DNA extraction step (Chai et al. 2016; Goldfarb et al. 2021; Jones et al. 2021).

### Extraction and amplification of DNA and sequencing of 16 S rRNA

The genomic DNA of the oral rinse and stool samples was extracted according to the manufacturer’s instructions using DNeasy PowerSoil Kit (Cat No: 47014, Qiagen, Germany). DNA concentration in each sample was measured using a Nanodrop spectrophotometer (ND-1000; Thermo Scientific, USA). The V3-V4 regions of the 16 S rRNA genes were amplified using the primers and Illumina adapter (underlined) listed below:

The forward primer was 5′ *TCGTCGGCAGCGTCAGATGTGTATAAGAGACAG*CCTACGGGNGGCWGCAG 3′, and the reverse primer was 5′ *GTCTCGTGGGCTCGGAGATGTGTATAAGAGACAG*GACTACHVGGGTATCTAATCC 3′. The molecular size and quality of the amplified products were examined using agarose gel electrophoresis (1%). Finally, libraries and PCR amplicons of the samples were prepared with IGA Technology Services (Udine, Italy) and sequenced using the Illumina MiSeq Platform (Illumina, San Diego, CA). Negative extraction controls and PCR blanks were included in each batch and sequenced at the same time as the samples to confirm that no contamination occurred during DNA extraction or amplification.

### Data analysis and statistics

Amplicon sequence variants (ASVs) serve as the basis for analyzing and classifying raw 16 S rRNA reads. The raw reads were delivered primer- and adapter-trimmed by the sequencing provider; no additional trimming was needed. To preprocess the sequences, the raw data were imported into the Quantitative Insights into Microbial Ecology 2 platform (QIIME2). DADA2 software, integrated with QIIME2, was used to trim and filter out reads with a median Phred < 25, allowing for a maximum of two expected errors per read. Additionally, DADA2 was utilized to denoise the 16 S rRNA reads, with forward reads being truncated to a length of 270 base pairs (bp) and reverse reads being truncated to 210 bp. This process generated a feature table of high-resolution amplicon sequence variants (ASVs), as previously described in the literature (Callahan et al. 2016; El Menofy et al. 2022; Tawfik et al. 2023). Taxonomy was assigned with a naive Bayes classifier trained on SILVA v138 sequences trimmed to the V3–V4 region (QIIME 2 feature classifier) (Quast et al. 2012; Wang et al. 2007). QIIME2 scripts were used to perform microbial diversity analysis along the orointestinal axis on the basis of both intercommunity features and intracommunity features. The alpha diversity of the Orointestinal axis microbiomes was assessed using richness indices (observed species and Chao1) and the Shannon diversity index for evenness. Permutational Multivariate Analysis of Variance (PERMANOVA) (Adonis R, Vegan package) was used to assess differences in bacterial community composition in relation to lifestyles and anatomical sites on the basis of both unweighted and weighted UniFrac distance matrices (Anderson 2001). Genus-level relative abundances were compared across groups using Kruskal-Wallis tests with Benjamini-Hochberg FDR correction. For genera showing significant overall differences (*p.adj* < 0.05) or those of a priori interest, pairwise comparisons were performed using Wilcoxon rank-sum tests with FDR correction. Effect sizes were calculated using Cohen’s d, with values > 0.5 considered large (Benjamini and Hochberg 1995). Tax4Fun was employed to infer the functional potential of the orointestinal microbiome (Aßhauer et al. 2015). The high-resolution amplicon sequence variant (ASV) table was used as input. The workflow included the following steps: (1) normalization of the ASV abundance table by the predicted 16 S rRNA copy number, (2) prediction of Kyoto Encyclopedia of Genes and Genomes (KEGG) Orthology (KO) abundances, and (3) aggregation of KO abundances into KEGG pathways (Kanehisa and Goto 2000). DESeq2 was applied to unrarefied integer count tables (aggregated to genus) and to predict KO profiles between groups in the dataset (FDR-corrected p value 0.05) to identify the differentially represented genera and KOs that generated the shifts in microbiomes (Love et al. 2014). The co-occurrence networks of microbial genera were generated using the SPIEC-EASI algorithm with the Meinshausen–Bühlmann method (Kurtz et al. 2015). The following parameters were utilized: lambda.min.ratio = 0.01, nlambda = 20, and pulsar threshold = 0.05. From the resulting networks, edge colors represent association strength, with a blue–purple–orange gradient indicating weak to strong correlations. The top 25 genera by degree centrality were visualized for each group. Hub taxa were identified as nodes with degree centrality above the 90th percentile within each network. All networks were analyzed separately for each disease group using the igraph package (Csardi 2013). Co-occurrence networks were also constructed based on Spearman correlation analysis. Only robust correlations with a strength of *r* ≥ 0.6 and a significance of *p* < 0.05 (after FDR correction) were considered for network visualization and interpretation (El Menofy et al. 2022; Tawfik et al. 2023). To comprehensively characterize taxonomic shifts, we employed two complementary differential abundance methods: LEfSe, which identifies biomarkers based on effect size and statistical significance while considering biological consistency, and DESeq2, which provides precise quantification of fold changes with rigorous FDR correction. Together, these approaches offer both discriminative biomarkers and quantitative effect sizes. LEfSe was employed to predict potential biomarkers linked with health states and anatomical sites (LDA scores > 2.0, *p* < 0.05) (Segata et al. 2011). Enterotyping was performed by partitioning around medoids (PAM) clustering on the basis of Jensen–Shannon divergence distances derived from genus-level relative abundances. The optimal number of clusters was identified using both the silhouette width and the Calinski-Harabasz index (Arumugam et al. 2011). Machine learning techniques have been employed and validated for microbiome research to identify microbial signatures distinguishing Obese-Smokers from matched groups (Pasolli et al. 2016). Using genus-level abundance data as input, we implemented a random forest model (randomForest package) configured with 500 decision trees and a maximum depth of 5. To define key bacterial discriminators, we additionally applied the Random Forest classifier via MicrobiomeAnalyst (Dhariwal et al. 2017; Knights et al. 2011). Model validation comprised (1) nested cross-validation (5 outer folds, 10 inner folds) evaluating the AUC, precision-recall, and calibration; (2) independent testing on holdout samples (30% of the data); and (3) 1000 permutation runs to confirm robustness. All plots and statistical tests were performed using R packages (version 4.4.2) (R Core Team 2021).

## Results

### Patient characteristics

The cohort comprised 108 participants stratified equally into groups (*n* = 27/group): healthy Non-Obese Non-Smokers (Control), Obese Non-Smokers, Non-Obese Smokers, and Obese-Smokers. Groups were age- and sex-matched (*p* > 0.40). As expected, Obese individuals and Obese-Smokers exhibited significantly greater BMIs and waist circumferences (*p* < 0.001) (Table 1). Among Obese-Smokers, metabolic dysfunction was most severe, as evidenced by elevated CRP (5.2 ± 1.8 mg/L vs. 1.2 ± 0.5 in controls, *p* < 0.001) and increased HOMA-IR (4.1 ± 0.9 vs. 1.1 ± 0.3, *p* < 0.001). Smokers reported substantial pack-year exposure (Obese-Smokers: 22.4 ± 6.8; Smokers: 18.7 ± 5.3). Obese-Smokers reported the highest cigarette consumption (18.9 ± 4.1/day), which exceeded that reported by smokers (14.2 ± 3.8/day, *p =* 0.002).

**Table 1:** Patient characteristics by groups

Characteristic	Control (*n* = 27)	Obese individuals (*n* = 27)	Smoker (*n* = 27)	Obese-Smoker (*n* = 27)	*p* value
Age (years), mean ± SD	45.2 ± 8.7	46.8 ± 9.1	47.3 ± 7.9	48.6 ± 8.5	0.42
Male sex, n (%)	14 (51.9%)	15 (55.6%)	16 (59.3%)	17 (63.0%)	0.78
BMI (kg/m^2^), mean ± SD	22.4 ± 1.8	34.7 ± 3.2*	23.1 ± 2.1	35.9 ± 4.1*†	< 0.001
Waist circumference (cm)	82.3 ± 6.5	112.6 ± 8.9*	84.1 ± 7.2	115.8 ± 9.3*†	< 0.001
Cigarettes/day, mean ± SD	0	0	14.2 ± 3.8*	18.9 ± 4.1*†	< 0.001
Current smoker, n (%)	0 (0%)	0 (0%)	27 (100%)*	27 (100%)*	< 0.001
CRP (mg/L), mean ± SD	1.2 ± 0.5	3.8 ± 1.1*	2.3 ± 0.9*	5.2 ± 1.8*†‡	< 0.001
HOMA-IR, mean ± SD	1.1 ± 0.3	3.2 ± 0.7*	1.6 ± 0.4*	4.1 ± 0.9*†‡	< 0.001
Fasting glucose (mg/dL)	92.3 ± 6.8	108.7 ± 9.4*	95.1 ± 7.2	116.4 ± 11.2*†	< 0.001

*Values are presented as mean ± SD or n (%). BMI, body mass index; HOMA-IR, Homeostatic Model Assessment of Insulin Resistance; CRP, C-reactive protein. Statistical comparisons were performed using ANOVA for continuous variables and chi-square tests for categorical variables. Significantly different from the control group (*p* < 0.05); †significantly different from the smoking group (*p* < 0.05); ‡significantly different from the obese group (*p* < 0.05)

### Divergent alpha and beta diversity patterns in the orointestinal axis of smokers and obese individuals

**Fig. 2:**
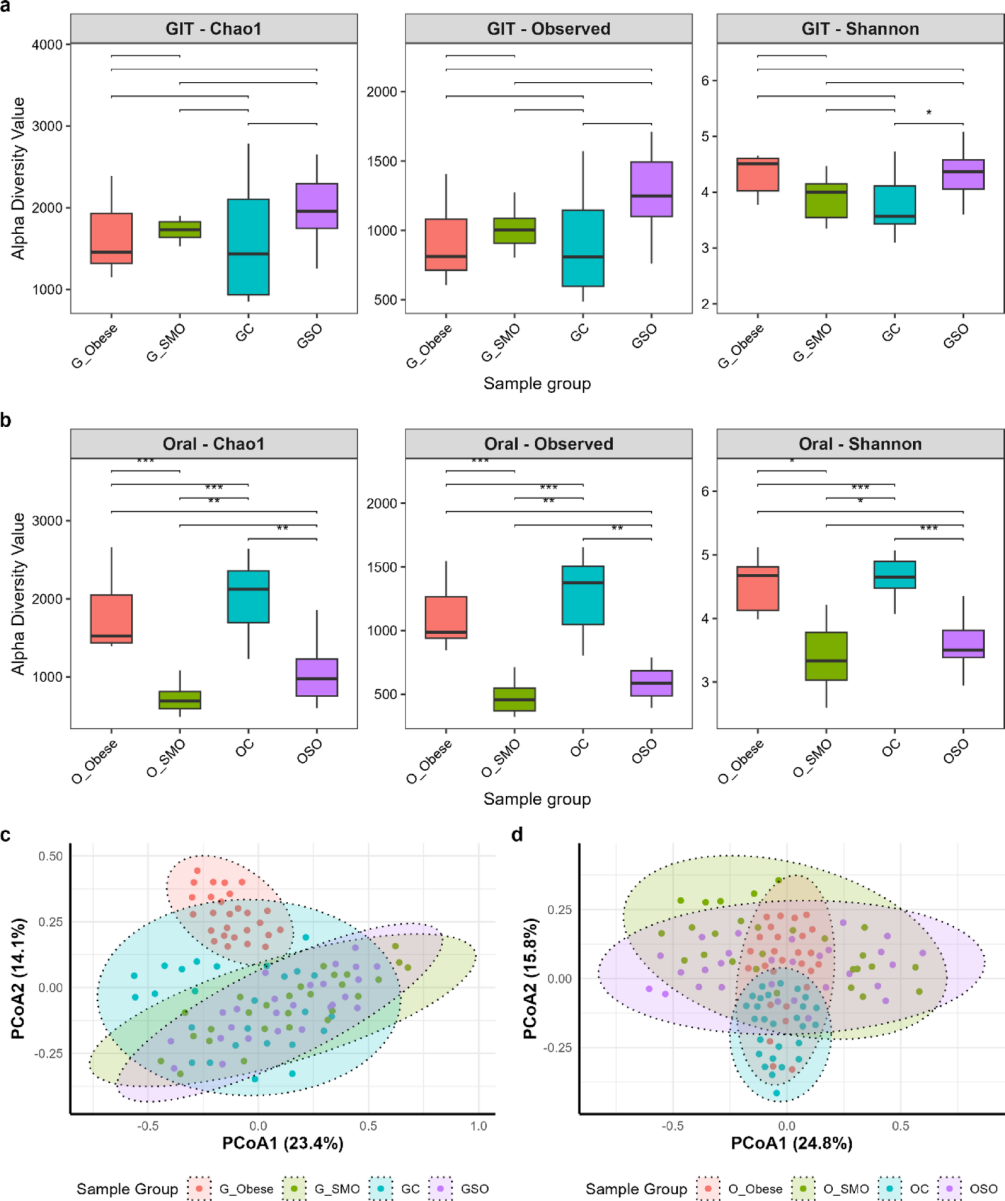
Alpha and beta diversity of the oral and gut microbiomes across the obesity and smoking groups. **a**,** b** Box plots showing alpha diversity indices (Chao1, observed species, and Shannon diversity) for the gut (a) and oral (b) microbiomes across the four groups. Significant pairwise differences are indicated by asterisks (*p* < 0.05, *p < 0.01, **p < 0.001). **c**,** d** Principal coordinate analysis (PCoA) plots based on weighted UniFrac distance matrices for the gut (c) and oral (d) microbiomes. Each point represents a sample, colored by group. The ellipses indicate 95% confidence intervals. G = Gut sample, O = Oral sample, SMO = Smokers, SO = Obese-Smokers, C = Control individuals, Obese = Obese individuals

The orointestinal axis revealed distinct microbial diversity patterns across obesity- and smoking-defined groups, with the oral microbiome showing more pronounced and statistically supported differences than the gut microbiome (Fig. 2).

Analysis of the gut microbiome indicated overall stability in alpha diversity across groups, as pairwise comparisons of gut alpha diversity metrics did not yield statistically significant differences (*p* ≥ 0.05) (Fig. 2a). In stark contrast, all alpha diversity metrics for the oral microbiome (Chao1, observed species, and Shannon indices) revealed significant pairwise differences between groups (Fig. 2b). Compared with Smokers and Obese-Smokers, Controls presented significantly greater richness and evenness. Specifically, Chao1 richness was significantly lower in smokers (*padj* = 0.000524) and Obese-Smokers (Wilcoxon test; *padj* = 0.004) than in controls. Similarly, Obese individuals presented higher Chao1 values than did both smokers (*padj* = 0.000933) and Obese-Smokers (*padj* = 0.006). These patterns were consistent for the observed species indices. Shannon diversity was also significantly lower in Smokers and Obese-Smokers than in controls (Kruskal‒Wallis; *padj* = 0.002). A dose-dependent effect of smoking was evident, with heavy smokers (> 20 cigarettes/day) exhibiting 34% lower oral Shannon diversity than light smokers (< 10 cigarettes/day; *p =* 0.008).

Distinct compartment-specific responses were observed: while heavy smoking was associated with markedly reduced oral alpha diversity, gut microbial diversity remained largely stable across groups, except for the specific reduction in Shannon diversity among smokers, despite significant beta diversity shifts. PERMANOVA confirmed significant overall compositional differences in both the gut (F = 1.9891, R^2^ = 0.181, *p =* 0.003) and oral (F = 1.6644, R^2^ = 0.1611, *p =* 0.023) microbiomes (Fig. 2c, d).

### Taxonomic profiling and phylum-level composition

**Fig. 3:**
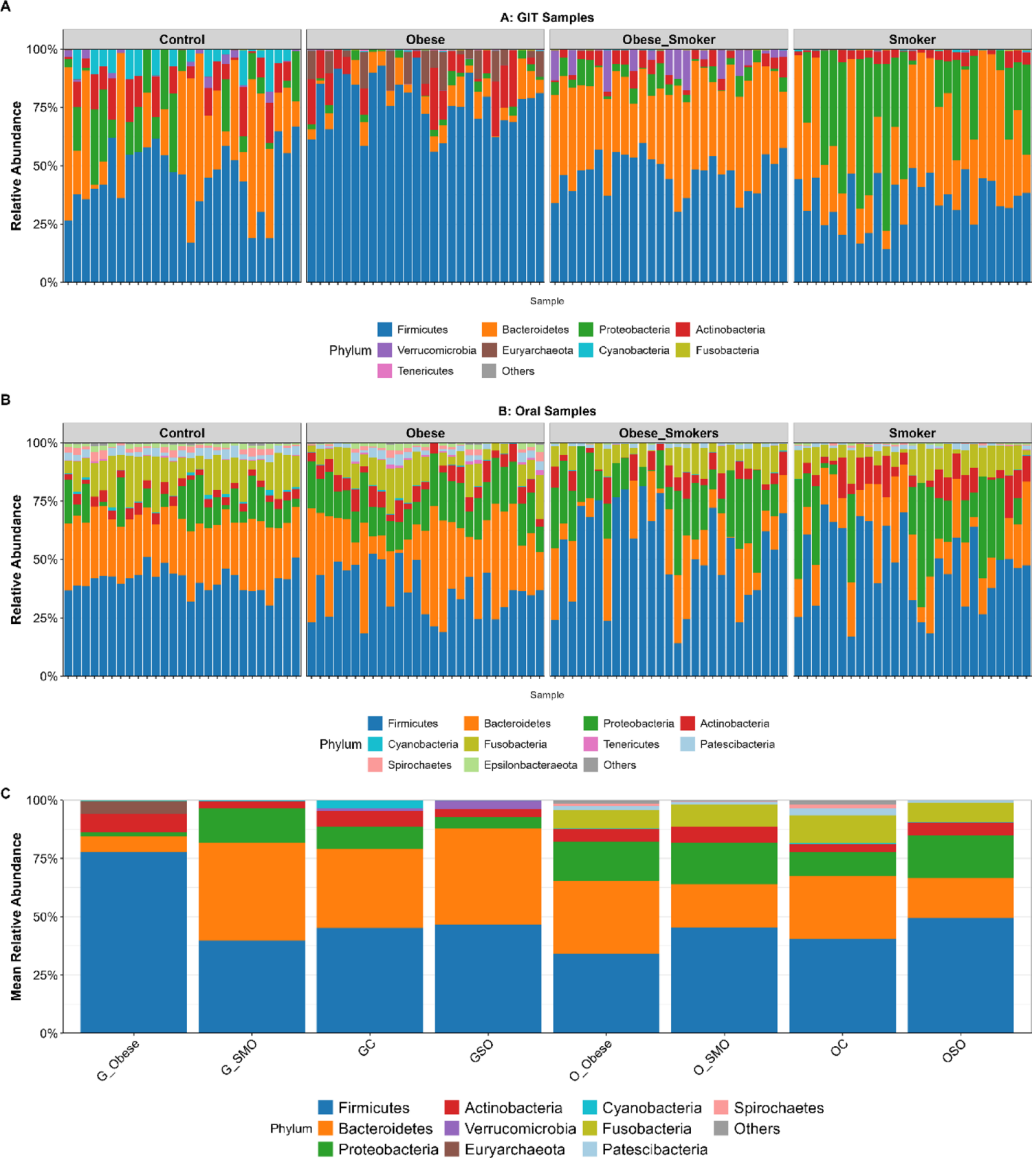
Per-participant phylum-level composition of oral and gut microbiota across clinical groups. **A** Gut microbiota: each bar represents an individual stool sample (*n* = 27 per group). **B** Oral microbiota: each bar represents an individual oral rinse sample (*n* = 27 per group). Colors indicate relative abundance of the top 10 phyla; rare phyla are grouped as ‘Others’. Facets separate the four clinical groups: Control, Obese, Smoker, and Obese-Smoker. **C** Group-mean comparisons of oral and gut microbiota

Following sequencing, a total of 7,331,962 raw reads were obtained across all samples. After preprocessing, including quality filtering, denoising, and removal of chimeric sequences, a high-quality dataset comprising 6,356,811 reads was retained, with an average of 35,072 ± 4831 reads per sample. The sequenced extraction and PCR blanks presented negligible read counts with no recurrent taxa; the blanks were monitored to rule out reagent contamination. The rarefaction depth of 26,078 reads was used for downstream analyses (Additional Figure S1). A comprehensive taxonomic analysis of 108 orointestinal microbiomes, which included 29 unique phyla, 64 classes, 135 orders, 315 families, 938 genera, and 2672 species, revealed significant phylum-level alterations across obesity- and smoking-defined groups (Kruskal‒Wallis; H = 18.42, *p =* 0.0026; Fig. 3). Clear compositional differences were observed between oral and gut samples, with patterns varying according to clinical status. In the gut microbiome, the abundance of Bacteroidetes was comparable between controls (50.1 ± 11.8%) and Obese-Smokers (37.7 ± 6.2%) but was significantly depleted in Smokers (28.6 ± 15.1%; *p =* 0.003). Firmicutes abundance in the gut remained stable across groups (*p =* 0.12). The most dramatic shift occurred in Proteobacteria, which doubled in Obese-Smokers’ gut samples (*p =* 0.008). Conversely, in the oral microbiome, Smokers exhibited a significant depletion of Firmicutes (*p =* 0.04) and a tripling of Proteobacteria (*p =* 0.001). Furthermore, Spirochaetes, which were undetectable in controls, emerged and were significantly enriched in the oral samples of Obese-Smokers compared with those of Controls (Wilcoxon, *p =* 0.007). 

### Firmicutes/bacteroidetes (F/B) ratio dynamics

The F/B ratio exhibited distinct compartment- and phenotype-specific patterns. In the gut microbiome, Smokers showed the highest F/B ratio (1.78 ± 1.05), which was significantly greater than that of controls (0.91 ± 0.31; *p =* 0.006) and Obese-Smokers (1.33 ± 0.46; *p =* 0.04). Oral microbiome F/B ratios demonstrated smoking-associated increases (Smokers: 1.65 ± 0.65 vs. Controls: 1.45 ± 0.47; *p =* 0.03) but no obesity effect (Obese-Smokers: 1.42 ± 0.19 vs. Smokers; *p =* 0.41). Strikingly, the gut F/B ratio strongly correlated with metabolic dysfunction markers: it was positively correlated with CRP (*r =* 0.71, *p* < 0.001) and HOMA-IR (*r =* 0.63, *p =* 0.002) and negatively correlated with Bacteroidetes abundance (*r =* -0.82, *p* < 0.001). The oral F/B ratios showed weaker clinical correlations but strong smoking dose dependence (*r =* 0.67 with cigarettes/day, *p =* 0.001).

### Differential abundance and biomarker identification in the orointestinal axis: synergistic effects of obesity and smoking

**Fig. 4:**
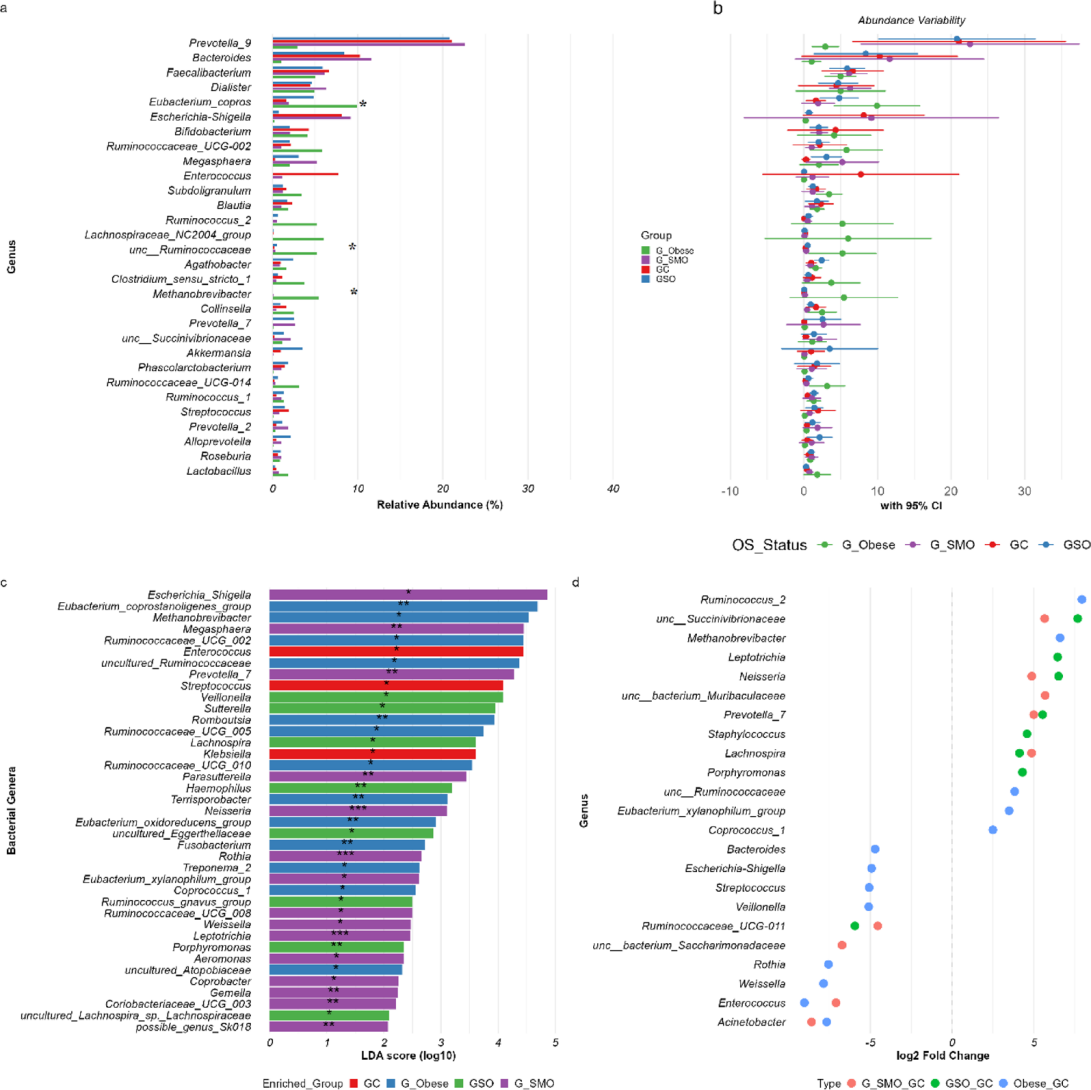
Differential abundance and biomarker analysis of gut bacterial genera across clinical groups. Multi-panel visualization integrating complementary analytical approaches. **a** Mean relative abundance (%) of the top 30 genera, with groups color-coded as indicated. Statistical significance is measured by the Kruskal-Wallis rank-sum test, is indicated directly by * *p.adj* < 0.05, ** *p.adj* < 0.01). **b** 95% confidence intervals for genus-level abundances, illustrating within-group variability. **c** LEfSe analysis identifying genera with significant discriminatory power (LDA score > 2.0, *p < 0.05) as potential biomarkers for each group. Bar length represents log₁₀ LDA score. Asterisks indicate statistical significance: *p < 0.05, **p < 0.01, ***p < 0.001. **d** DESeq2-derived log_2_ fold changes for the top 30 significantly differentially abundant genera between groups. Dot size reflects -log₁₀(*padj*). All p-values were FDR-corrected for multiple comparisons. G = Gut sample, O = Oral sample, SMO = Smokers, SO = Obese-Smokers, C = Control individuals, Obese = Obese individuals

In the gut microbiome, Significant differences were observed for *Eubacterium coprostanoligenes* group (*p.adj* = 0.042 for Obese vs. Smokers and Obese vs. Controls), *Methanobrevibacter* (*p.adj* = 0.016 for Obese vs. Obese-Smokers; *p.adj* = 0.042 for Obese vs. Smokers), and uncultured *Ruminococcaceae* (*p.adj* = 0.022 for Obese vs. Controls; *p.adj* = 0.033 for Obese vs. Smokers). These genera were significantly enriched in Obese individuals with large effect sizes (effsize > 0.6) (Fig. 4). Furthermore, dominant genera such as *Bacteroides*, *Escherichia_Shigella*, *Ruminococcus_2*, *Eubacterium_coprostanoligenes_group*, *Enterococcus*, *Streptococcus*, *Methanobrevibacter*, *Veillonella*, and *Sutterella* were most significantly perturbed by obesity and smoking, as confirmed by both differential abundance (DESeq2) and biomarker enrichment (LEfSe) analyses (Fig. 4). DESeq2 revealed pronounced depletion of *Bacteroides* (log_2_FC = − 4.70, *padj* = 0.0074) and *Escherichia_Shigella* (log_2_FC = − 4.92, *padj* = 3.8 × 10^−5^) in Obese individuals, alongside losses of *Enterococcus* (log_2_FC = − 9.02, *padj* = 7.9 × 10^−5^) and *Streptococcus* (log_2_FC = − 5.07, *padj* = 1.2 × 10^−5^). Conversely, *Ruminococcus_2* (log_2_FC = + 7.94, *padj* = 6.9 × 10^−6^), *Methanobrevibacter* (log_2_FC = + 6.60, *padj* = 0.0011), and *Eubacterium_coprostanoligenes_group* (log_2_FC = + 2.95, *padj* = 0.042) were enriched in obesity. LEfSe further validated these patterns, identifying *Escherichia_Shigella* (LDA = 4.86, *p =* 0.029) and *Prevotella_7* (LDA = 4.28, *p =* 0.008) as smoker-enriched pathobionts, whereas *Eubacterium_coprostanoligenes_group* (LDA = 4.69, *p =* 0.009) and *Methanobrevibacter* (LDA = 4.53, *p =* 0.027) were obesity-associated biomarkers. Smoking amplified dysbiosis, depleting *Escherichia_Shigella* (log_2_FC = − 2.78, *padj* = 0.044) and *Enterococcus* (log_2_FC = − 7.09, *padj* = 0.0023) in Obese-Smokers, while enriching Orointestinal ecological linkages such as *Neisseria* (LDA = 3.10, *p =* 2.18e−05; log_2_FC = + 4.87, *padj* = 9.8 × 10^−6^) and *Veillonella* (LDA = 4.08, *p =* 0.023).

**Fig. 5:**
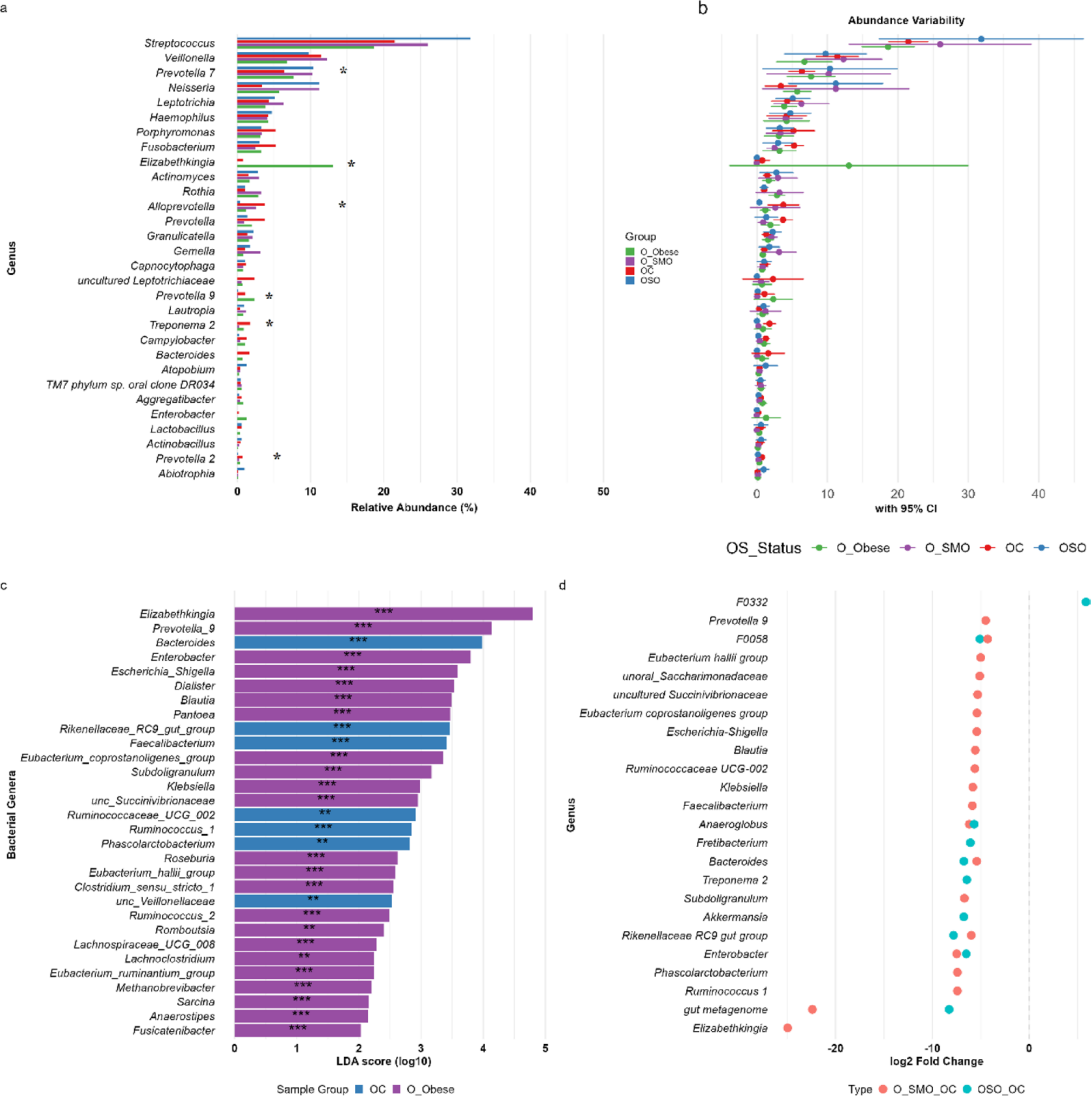
Differential abundance and biomarker analysis of oral bacterial genera across clinical groups. Multi-panel visualization integrating complementary analytical approaches. **a** Mean relative abundance (%) of the top 30 genera, with groups color-coded as indicated. Statistical significance is measured by Kruskal-Wallis rank-sum test, and is indicated directly by * *p.adj* < 0.05, ** *p.adj* < 0.01). **b** 95% confidence intervals for genus-level abundances, illustrating within-group variability. **c** LEfSe analysis identifying genera with significant discriminatory power (LDA score > 2.0, *p < 0.05) as potential biomarkers for each group. Bar length represents log₁₀ LDA score. Asterisks indicate statistical significance: *p < 0.05, **p < 0.01, ***p < 0.001. **d** DESeq2-derived log_2_ fold changes for the top 30 significantly differentially abundant genera between groups. Dot size reflects -log₁₀(*padj*). All p-values were FDR-corrected for multiple comparisons. G = Gut sample, O = Oral sample, SMO = Smokers, SO = Obese-Smokers, C = Control individuals, Obese = Obese individuals

In the oral microbiome, key taxa, including *Streptococcus*, *Veillonella*, *Prevotella_7*, *Neisseria*, *Haemophilus*, *Porphyromonas*, *Elizabethkingia*, *Fusobacterium*, and *Alloprevotella*, showed robust alterations across both analytical methods (Fig. 5). Kruskal-Wallis analysis revealed significant overall differences for *Prevotella* 9 (*p.adj* = 0.017). Pairwise comparisons identified significant depletion of *Alloprevotella* in Obese-Smokers vs. Controls (*p.adj* = 0.002) and Obese vs. Controls (*p.adj* = 0.033); enrichment of *Prevotella* 9 in Obese vs. Obese-Smokers (*p.adj* = 0.007) and Obese vs. Smokers (*p.adj* = 0.008); depletion of *Treponema* 2 in Obese-Smokers vs. Controls (*p.adj* = 0.007) and vs. Smokers (*p.adj* = 0.029); depletion of *Bacteroides* in Obese-Smokers vs. Obese (*p.adj* = 0.042) and vs. Controls (*p.adj* = 0.042); depletion of *Campylobacter* in Obese-Smokers vs. Controls (*p.adj* = 0.042); and enrichment of *Fusobacterium* in Smokers vs. Controls (*p.adj* = 0.022). All significant comparisons showed large effect sizes (effsize > 0.6). DESeq2 revealed profound depletion of commensals in Obese-Smokers, including *Elizabethkingia* (log_2_FC = − 6.33, *padj* = 0.0032), *Bacteroides* (log_2_FC = − 6.75, *padj* = 8.2 × 10^−5^), *Alloprevotella* (log_2_FC = − 3.67, *padj* = 0.0030), and *Fretibacterium* (log_2_FC = − 6.07, *padj* = 0.0006). LEfSe reinforced these findings, highlighting *Elizabethkingia* (LDA = 4.8, *p =* 4.90e-05) and *Alloprevotella* (LDA = 4.42, *p =* 0.007) as biomarkers for nonsmoker groups, whereas *Escherichia_Shigella* (LDA = 3.59, *p =* 0.0002) and *Klebsiella* (LDA = 2.98, *p =* 3.75e-05) were enriched in Obese individuals. Smokers presented unique signatures, such as *Vibrio* (LDA = 2.39, *p =* 0.042) and *Olsenella* (LDA = 2.26, *p =* 0.035), whereas Obese-Smokers showed depletion of keystone taxa (*Treponema_2*, *Campylobacter*, *Faecalibacterium*) and enrichment of *Lactobacillus* (LDA = 3.66, *p =* 0.02). This convergent oral dysbiosis, characterized by loss of protective commensals and expansion of pathobionts, underscores the synergistic impact of obesity and smoking on microbial ecology.

The application of machine learning identified highly specific microbial biomarkers capable of distinguishing patient groups with high accuracy, underscoring the diagnostic potential of the orointestinal microbiome. The most striking signature was found in the oral cavity, where the genus *F0058* (Saccharimonadaceae) was associated with the Obese-Smoker status with exceptional precision (AUC = 0.975, *padj* = 0.017) (Additional Figures S2 and S5). This potential oral biomarker significantly outperformed gut-derived signals. In the gut microbiome, the pathobiont *Escherichia_Shigella* was the most robust discriminator for the Obese group (AUC = 0.851, *padj =* 0.0038). In contrast, *Ruminococcaceae UCG-010* achieved the highest predictive power for identifying Obese-Smokers from controls (AUC = 0.905, *p =* 0.0014) (Additional Figures S3 and S4). Furthermore, the oral pathobiont *Neisseria* was a strong classifier for the Smoker group (AUC = 0.842, *padj =* 0.0037).

### Gut and oral enterotypes define an orointestinal axis of dysbiosis

Enterotyping analysis revealed three distinct gut microbial enterotypes that stratify closely according to clinical status (Additional file; Table S1). A Bacteroides-driven enterotype, predominant in healthy controls, was characterized by high abundances of *Bacteroides*, *Faecalibacterium*, *Megasphaera*, *Collinsella*, and *Ruminococcus_1*. This community was enriched with short-chain fatty acid (SCFA) producers and presented a low abundance of methanogens and Proteobacteria (Kruskal-Wallis, *p =* 0.006 for *Bacteroides*). In contrast, lean smokers were characterized by a Prevotella-dominated enterotype (*Prevotella_9*, *Prevotella_7*, *Phascolarctobacterium*, *Alloprevotella*, *Dialister*), which featured intermediate SCFA levels, elevated proinflammatory LPS producers, and significant depletion of *Bacteroides* (*p =* 0.003). The most dysbiotic profile, a methanogenic- and pathobiont-rich enterotype, was most common in Obese-Smokers. Marked by enrichment of *Ruminococcus_2*, *Methanobrevibacter*, *Eubacterium_copros*, *Lachnospiraceae_UCG-008*, and *Klebsiella*.

Analogous patterns were observed in the oral microbiome, forming three corresponding enterotypes that mirror systemic exposures (Additional file; Table S2). The controls presented a balanced *Streptococcus–Haemophilus* enterotype (*Streptococcus*, *Haemophilus*, *Veillonella*, *Rothia*, *Granulicatella*) with high microbial stability and minimal pathobionts (*p =* 0.008 for *Streptococcus* abundance). Smokers were enriched in a mixed *Veillonella–Neisseria* assemblage, which presented moderate SCFA production but increased Proteobacteria and a loss of protective commensals such as *Elizabethkingia* (*p =* 0.010). The most severe oral dysbiosis was found in Obese-Smokers, who coalesced into a pathobiome-rich *Neisseria -Fusobacterium* enterotype (*Neisseria*, *Fusobacterium*, *Porphyromonas*, *Treponema_2*, *Campylobacter*), which carried the highest pathobiont load (*p =* 0.005) and was strongly correlated with clinical inflammatory markers.

### Compartment-specific network architectures reveal microbial ecological strategies

We utilized SPIEC-EASI to investigate bacterial interactions and evaluate potential variations in the organization of microbial communities between the different studied groups for both the gut and oral microbiomes. The architecture of the microbial co-occurrence networks revealed starkly different ecological strategies between the oral and gut compartments under stress.

The disturbed gut microbiome was structured by intense antagonism between commensal and pathogenic taxa (Additional file; Table S3). The Controls exhibited a stable and complex structure with more connected components (*n* = 3), a greater average path length (6.12), and greater modularity (0.71), indicating a resilient and compartmentalized community governed by 61 hub taxa, such as *Lachnospiraceae UCG-006* (Degree = 6). In Smokers, a powerful mutualism between the beneficial genera *Ruminococcus_1* (Degree = 4 in GC) and *Ruminococcaceae UCG-002* (Degree = 4 in GC) (*r =* 0.88) was directly opposed by strong negative correlations with the pathobiont *Enterobacter* (*r = *− 0.98) (Additional file; Table S4). The Obese individuals’ gut featured a unique synergistic relationship between *Methanobrevibacter* (Degree = 6) and *Romboutsia* (Degree = 4) (*r =* 0.89), suggesting an adaptive strategy for enhanced energy harvest, which cooccurred with disruptive relationships such as *Lactobacillus* with *Faecalibacterium* (*r = *− 0.90). The gut of Obese-Smokers reinforced certain mutualisms (*Alistipes* with *Barnesiella*, *r =* 0.85) with the highest number of hub taxa (*n* = 65), indicating a highly interconnected but potentially unstable state of dysbiosis, featuring a mix of commensals and pathobionts such as *Bilophila* (Degree = 7).

**Fig. 6:**
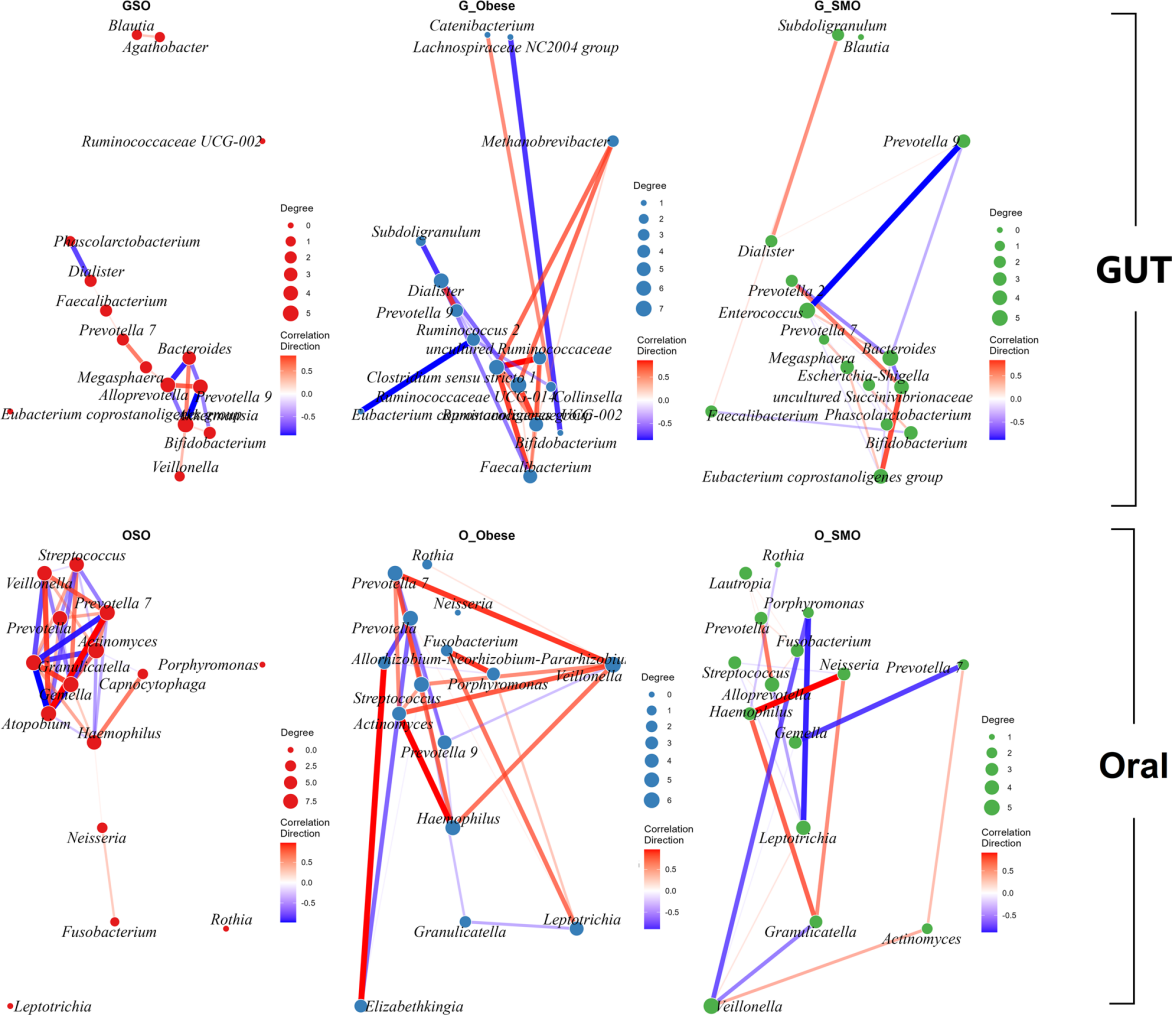
Gut microbial co-occurrence networks stratified by obesity and smoking status. Co-occurrence networks were constructed for the oral and gut microbiomes of participants. Networks depict Spearman correlations among the top 15 genera in each group. Nodes represent bacterial genera and are scaled by degree centrality, indicating the number of significant co-occurrences per genus. Node color denotes group identity (red, Obese smokers; blue, Obese; green, Smokers). Edges represent pairwise correlations with *r* ≥ 0.6; edge color reflects correlation direction (blue, negative; red, positive). G = Gut sample, O = Oral sample, SMO = Smokers, SO = Obese-Smokers, C = Control individuals, Obese = Obese individuals

Conversely, in the oral microbiome, dysbiosis was characterized by the formation of highly cohesive, cooperative pathobiont consortia (Additional file; Table S5). The Controls exhibited a highly connected and efficient network architecture, characterized by the highest edge density (0.023), the shortest average path length (3.60), and the lowest modularity (0.50), maintained by a diverse suite of 50 hub taxa, including classic oral commensals and specialists such as *Candidatus Saccharibacteria oral taxon TM7x* (Degree = 10) and *Streptococcus* (Degree = 9) (Additional file; Table S6). In Obese individuals, a classic periodontal complex emerged, defined by a positive co-occurrence between *Prevotella* and *Campylobacter* (*r =* 0.93) embedded within a network including *Fusobacterium* and *Treponema_2* (*r =* 0.83). Smokers exhibited a different but equally robust consortium dominated by a strong partnership between *Aggregatibacter* (Degree = 8) and *Alloprevotella* (Degree = 9) (*r =* 0.80) (Fig. 6). The most severe dysbiosis was observed in Obese-Smokers, where the network topology shifted from cooperation to extreme antagonism, as evidenced by near-perfect negative correlations (*Atopobium* with *Granulicatella*, *r = *− 0.98), indicating competitive exclusion and an altered network structure consistent with a diseased state. This was reflected in its severely disrupted network, which was fragmented into 26 connected components (LCC relative size = 0.264), with the lowest clustering coefficient (0.047) and highest modularity (0.88).

### Orointestinal axis disruption and linking in obese smokers

In healthy controls, the oral and gut microbiomes are ecologically distinct, with minimal covariation for shared genera (all *r* < 0.60), which could reveal independent community structures maintained by effective physiological barriers. Crucially, our results demonstrate parallel yet distinct dysbiosis across the oral-gut axis in response to smoking and obesity. The most severe ecological disruption was consistently observed in the Obese-Smokers plots at both sites. The gut Obese-Smokers responded with hyperconnectivity and a surge in hub taxa (*n* = 65). Conversely, the oral cavity of Obese-Smokers exhibited extreme fragmentation (26 components) and connectivity loss, indicating ecological linkage.

### Inferred functional metagenomic profiling reveals pathway-level disruptions

Functional prediction using Tax4Fun based on 16 S rRNA data revealed significant alterations in the genetic potential of the orointestinal microbiome (Additional Figures S6 and S7). In the gut microbiome of Obese-Smokers, we observed a significant depletion of KOs related to butyrate kinase (K00929) and butyrate-acetoacetate CoA-transferase (K01034), key enzymes for butyrate synthesis (log_2_FC = − 1.95, *padj* = 0.012). This coincided with the reduction in SCFA producers such as *Bacteroides* and *Faecalibacterium*. Conversely, KOs involved in LPS biosynthesis (K02527 and K02777) were markedly enriched (log_2_FC = + 1.72, *padj* = 0.007), aligning with the expansion of Proteobacteria. In the oral microbiome, a significant enrichment of KOs involved in glutathione metabolism (K00383, K00382) and superoxide dismutase (K04564) was detected in smokers and Obese-Smokers (log_2_FC = + 2.1, *padj* = 0.002), which could support a predicted increase in oxidative stress resistance. Furthermore, the co-occurrence of oral pathobionts was associated with inferred KOs for flagellar assembly (K02389, K02392) and type IV secretion systems (K03221).

## Discussion

Obesity and smoking cause over 12 million annual deaths worldwide. Our study identified novel orointestinal microbiome disruptions in comorbid individuals, revealing compartment-specific dysbiosis (Kumar et al. 2025; Wu et al. 2025).

Our cohort stratification revealed profound metabolic escalations directly linked to microbiome perturbations. Compared with controls, Obese-Smokers presented significantly elevated C-reactive protein levels and increased HOMA-IR, confirming synergistic inflammation and metabolic dysregulation (Pirih et al. 2021; Zhang et al. 2025). Oral *Elizabethkingia* depletion is correlated with elevated CRP levels (Duan et al. 2025; Zdziarski et al. 2017). Smoking intensity is correlated with oral *Neisseria* enrichment and covariation across habitats, whereas body mass index inversely tracks gut *Bacteroides* abundance, implicating the host phenotype as a microbiome modulator (Leite et al. 2022; Shapiro et al. 2022). These trends suggest that obesity and smoking may interact to intensify microbial and metabolic disturbances (Duan et al. 2025; Eva et al. 2025). The consistency of these patterns across diverse demographic subgroups underscores their biological importance and clinical relevance for risk stratification (Shapiro et al. 2022). Oral diversity decreases with the number of cigarettes smoked/day, suggesting a threshold‑like response, which is consistent with smoke‑associated epithelial damage models (Kato et al. 2010; Upadhyay et al. 2023). The superiority of daily consumption over pack-years in predicting microbial shifts indicates that acute exposure is associated with pathobiont expansion, informing targeted interventions for heavy smokers (Pleasants et al. 2020).

Compared with those of controls, the combined effects of obesity and smoking resulted in increased alpha diversity in Smokers and Obese-Smokers compared to controls (Imade and Obayagbona 2024b; van Dijk et al. 2024). While oral microbiome diversity declined by 34% in heavy smokers (Chattopadhyay et al. 2024), gut communities maintained alpha diversity despite compositional shifts (Chattopadhyay et al. 2024). Crucially, oral dysbiosis preceded gut changes in most Obese-Smokers, positioning the mouth as a potential early indicator of systemic inflammation (Batatinha et al. 2019; Mehrotra et al. 2010). These compartment-specific responses highlight the unique vulnerability of the oral microbiome to environmental‒metabolic interactions and its potential utility in presymptomatic disease screening (Mei and Li 2022; Xiao et al. 2020).

The observed shifts in the gut microbiome, particularly the increase in the Firmicutes/Bacteroidetes (F/B) ratio in Smokers, align with established smoking-associated dysbiosis patterns (Yan et al. 2021). This ratio elevation in Smokers stemmed from Bacteroidetes depletion (28.6% vs. 50.1% in controls), whereas obese individuals presented attenuated dysbiosis (Leite et al. 2022; Shapiro et al. 2022). Notably, compared with Smokers, Obese-Smokers presented attenuated F/B ratios, which could be due to the partial counteraction of obesity to the dysbiotic effects of smoking through distinct ecological mechanisms (Duan et al. 2025). The strong correlation between the gut F/B ratio and metabolic markers underscores its clinical relevance as a biomarker of inflammation and insulin resistance in comorbid states (Petakh et al. 2023; Xiao and Zhao 2014).

The observed compartment-specific responses were striking: while the oral microbiome presented minimal F/B ratio changes, smoking drove significant Proteobacteria expansion in both habitats (Mohammed et al. 2024). This aligns with global studies linking Proteobacteria enrichment to inflammation and barrier dysfunction (Chopyk and Grakoui 2020; Zeng et al. 2017). The exclusive oral colonization by Spirochaetes in Smokers further highlights smoking’s habitat-selective effects, corroborating dose-dependent Spirochaete proliferation in smoker-exposed oral niches (Al-Marzooq et al. 2022). These phylum-level signatures demonstrate the differential susceptibility of the orointestinal axis to lifestyle factors (Baima et al. 2023).

Genus-specific alterations revealed keystone taxa collapse under combined stress. *Elizabethkingia* spp. is still an understudied pathogen with an intrinsic multidrug resistance phenotype that has been reported in several countries around the world, causing opportunistic infections with high mortality rates(Zajmi et al. 2022). *Elizabethkingia* spp. is profoundly depleted in obese-smoking individuals, reflecting extreme susceptibility to metabolic-environmental insults. Concurrently, pathobionts thrived: oral *Escherichia_Shigella* and shared taxa (*Neisseria*) showing cross‑site covariation dominated disease states, encoding virulence factors that could exacerbate inflammation (Bombin et al. 2022). In the gut, the depletion of Bacteroides and the associated reduction in butyrate production could compromise colonocyte energy metabolism (Singh et al. 2022). These shifts could disrupt the symbiotic relationship between the host and commensal microbes, which in turn enables inflammatory pathobionts to dominate when smoking and obesity perturb microbial homeostasis. The genus-level specificity underscores how discrete microbial functions contribute to system-wide pathophysiology when disrupted (Rajasekaran et al. 2024; Sarafidou et al. 2024).

Machine learning identified potential biomarkers for clinical cross‑site co‑variation. *F0058* (oral Saccharimonadaceae) has exceptional predictive power for Obese-Smokers status and is strongly correlated with mucosal inflammation markers such as calprotectin (Descamps et al. 2024). In the gut, the impacts of *Neisseria* and *Bacteroides* discriminating between smoking and obesity are more accurate than traditional anthropometric measures (Mohammed et al. 2024). These taxa outperform BMI in comorbidity risk stratification, offering noninvasive diagnostic tools. *F0058*’s predictive capacity likely stems from its association with oxidative stress pathways, a canonical smoking response, whereas *Neisseria*’s dual role as an oral resident and gut colonizer during barrier breach makes it a unique sentinel. Accordingly, these patterns could be applied as potential biomarkers for the early identification of high-risk patients (Cryan et al. 2019; Maki et al. 2022; Mohammed et al. 2024).

Co-occurrence networks revealed pathogenic alliances reshaping microbial ecology. In Obese-Smokers, *Escherichia_Shigella* and *Fusobacterium* form synergistic biofilms, increasing LPS biosynthesis and epithelial invasion (Beverly et al. 2025). The cross-habitat synchronization of *Neisseria* confirmed smoking-associated covariation across habitats. Conversely, health-associated mutualisms such as *Bacteroides*-*Faecalibacterium* disintegrate in disease states, impairing butyrate production (Beverly et al. 2025). This reorganization mirrors that of the inflammatory bacteria in colorectal cancer, where pathobionts coopt mucosal resources while evading immune surveillance (Han et al. 2023). Network topology analysis revealed increased modularity in Obese-Smokers microbiomes, which might support rigid, pathogen-dominated communities resistant to dietary or probiotic interventions (Tapsell et al. 2016).

Enterotyping stratified the gut communities into three distinct metabolic phenotypes with clinical implications. The Proteobacteria-dominated enterotype (common in Obese-Smokers) was characterized by the presence of oral pathobionts (*Neisseria* and *Leptotrichia*) and was strongly correlated with insulin resistance. In contrast, the Bacteroides-driven enterotype was associated with normoglycemia, whereas the Prevotella-enriched cluster indicated a state of moderate inflammation. Enterotype stability differed across groups: Obese-Smokers exhibited rigid clustering. These configurations resemble the dysbiotic patterns observed in type 2 diabetes, where Proteobacteria expansion is predictive of therapeutic resistance, which might suggest a distinct and potentially entrenched dysbiotic profile (Fernandes et al. 2019; Neri-Rosario et al. 2023; Zhang et al. 2021). The enterotype framework thus offers a valuable microbial stratification system for the precise management of obesity-smoking comorbidities (Wu et al. 2025). Our findings further revealed that lean nonsmokers harbor a Bacteroides-rich, SCFA producer*-*dominated gut community alongside a *Streptococcus-Haemophilus* oral ecosystem, whereas Obese-Smokers exhibit methanogenic *Ruminococcus*/*Methanobrevibacter* and pathobiome-rich *Neisseria*/*Fusobacterium* profiles. This orointestinal coupling underscores the potential for targeted interventions, such as barrier reinforcement or probiotic restoration of keystone taxa, that may help disrupt the dysbiotic cycle.

The key genus that tracks disease markers with striking precision across the obesity and smoking groups is oral *Elizabethkingia*, which is inversely correlated with systemic inflammation, confirming its role in mucosal homeostasis (Zajmi et al. 2022; Zdziarski et al. 2017). Notably, *Fusobacterium* enrichment in Obese-Smokers correlated with histologically confirmed epithelial damage, suggesting direct pathobiont-mediated injury. Multivariate modeling revealed that microbial signatures explained 34% of the metabolic variance beyond traditional risk factors, highlighting their clinical utility for prognostication and personalized interventions (Kim et al. 2020; Queen et al. 2025; Shoji et al. 2021).

Our inferred functional profiling revealed two convergent patterns with potential clinical relevance. First, the depletion of butyrate synthesis KOs (K00929, K01034) in Obese-Smokers suggests a reduced capacity for butyrate production. Butyrate is essential for maintaining colonic epithelial integrity and regulating glucose metabolism (Singh et al. 2022; van Deuren et al. 2022); thus, this inferred deficit may contribute to the elevated HOMA-IR and CRP observed in this group (Table 1). Second, the enrichment of LPS biosynthesis KOs (K02777) aligns with the expansion of Proteobacteria and may reflect increased potential for endotoxin production, which could trigger systemic inflammation as evidenced by elevated CRP levels (Chopyk and Grakoui 2020; Zeng et al. 2017). These inferred functional shifts, reduced SCFA production and increased LPS biosynthesis capacity, are consistent with the “leaky gut” phenotype implicated in metabolic dysfunction (Chopyk and Grakoui 2020).

In the oral microbiome, enrichment of oxidative stress response KOs (K04564, K00383) in Smokers and Obese-Smokers suggests adaptation to cigarette smoke-derived oxidants, potentially selecting for pathobionts with enhanced stress resistance. This observation aligns with reports linking smoking to oxidative DNA damage in oral tissues (Cao et al. 2016).

We emphasize that these functional interpretations are based on inferred genetic potential from 16 S rRNA data, not direct measurements of metabolites, gene expression, or host immune responses. Nevertheless, they generate testable hypotheses for future mechanistic studies. For example, targeted metabolomics could quantify fecal butyrate concentrations, while LPS assays and inflammatory cytokine profiling could directly assess endotoxemia and immune activation. Such studies would determine whether the predicted functional deficits translate into the physiological consequences hypothesized here and would clarify whether the observed taxonomic shifts directly drive metabolic deterioration in Obese-Smokers.

The inferred upregulation of LPS biosynthesis and depletion of butyrate are associated with clinical markers and align with indicators of endotoxemia, which in turn are linked to increased CRP levels (Rajasekaran et al. 2024). Concurrent butyrate metabolism depletion impaired the energy supply of colonocytes, exacerbating insulin resistance (van Deuren et al. 2022). Oral communities in Smokers exhibit amplified oxidative stress responses, promoting DNA damage and carcinogenesis (Cao et al. 2016). The enrichment of LPS biosynthesis KOs, coupled with elevated CRP in Obese-Smokers, is consistent with the well-established role of LPS in triggering inflammatory responses via TLR4 (Huwart et al. 2024), though direct measurement of LPS and TLR4 activation was beyond the scope of this study. This functional convergence, despite habitat differences, could explain why Obese-Smokers exhibit accelerated metabolic decline compared with single-exposure groups, providing targets for pathway-specific therapeutics (Caliri et al. 2021; Kong et al. 2025).

Obesity and smoking jointly perturb the orointestinal axis, resulting in compartment-specific dysbiosis characterized by reduced SCFA production, depletion of beneficial commensals, and expansion of inflammatory pathobionts. Our findings align with those of Kumar et al. (2025), who reported gut Firmicutes depletion and Proteobacteria enrichment under metabolic stress (Kumar et al. 2025). We extend this understanding by showing that smoking is associated with coordinated covariation of genera across habitats, such as *Neisseria* and *Leptotrichia* (Duan et al. 2025), consistent with cross-site ecological structuring rather than microbial migration.

Nevertheless, our cross-sectional design precludes causal inference; longitudinal sampling during smoking cessation or weight loss interventions would clarify temporal dynamics (Imade and Obayagbona 2024a). While 16 S rRNA profiling limits species-level resolution and functional insight, our inferred LPS-biosynthesis upregulation echoes mechanistic models of dysbiosis, supporting inflammation-driven microbial shifts (Zeng et al. 2017). Dietary intake was not controlled, which may confound enterotype delineation; future metagenomic and metabolomic integration would strengthen functional attribution (Lloyd-Price et al. 2020; Pascal et al. 2017).

Functional predictions provide a potential mechanistic link between the observed taxonomic shifts and the clinical phenotype of metabolic dysfunction. The inferred depletion of butyrate synthesis pathways (K00929, K01034) suggests a potential reduction in butyrate production capacity, which is based on previous literature, may impair colonocyte energy metabolism (Calderaro et al. 2019). Its loss, coupled with the enriched LPS biosynthesis KOs (K02777), creates a perfect storm for a “leaky gut” phenotype and systemic endotoxemia, directly explaining the elevated inflammatory markers (CRPs) and insulin resistance (HOMA-IR) in the Obese-Smoker group (Chopyk and Grakoui 2020). Moreover, the increase in oxidative stress-resistant KOs (K04564, K00383) in the oral microbiome reflects a direct ecological response to the pro-oxidant chemicals in cigarette smoke, which selects for resistant pathobionts and damages the host mucosa (Chen et al. 2022; Huang and Shi 2019; Wirth et al. 2020). Finally, the coprediction of virulence-associated KOs (K02389, K03221) offers a plausible genomic mechanism for the covariation across habitats of oral taxa to the gut. These findings suggest that smoking and obesity fundamentally alter the functional capacity of the microbiome, driving it toward a more inflammatory and invasive state that exacerbates systemic disease. While these predictions are inferred from 16 S data, they provide a valuable, hypothesis-generating framework for future mechanistic studies.

These findings highlight the orointestinal axis as a key interface affected by the combined impact of obesity and smoking, where microbial covariation across habitats and loss of beneficial taxa may contribute to metabolic inflammation. Future studies should track microbiome dynamics during GLP-1 agonist therapy and validate inferred pathways such as LPS biosynthesis through metabolomics. Targeted restoration of orointestinal microbial homeostasis through barrier reinforcement or pathobiont suppression could mitigate the risk of obesity-smoking comorbidity.

The oral-gut microbiota axis has been increasingly implicated in obesity-related cardiovascular diseases, including hypertension, atherosclerosis, and myocardial infarction (López-Tenorio et al. 2026; Ruan et al. 2023; Verhaar et al. 2020). A recent study of ST-segment elevation myocardial infarction (STEMI) patients revealed compartment-specific responses strikingly similar to our findings: oral microbiome diversity was significantly altered while gut community structure remained stable, with oral bacteria potentially influencing gut SCFA-producing communities (Constantino-Jonapa et al. 2025). This convergence suggests that oral vulnerability with gut resilience may be a generalizable pattern in cardiometabolic disease. Microbial metabolites play opposing roles in cardiovascular pathogenesis, SCFAs exert protective effects through immune modulation and barrier maintenance, while TMAO promotes inflammation, endothelial dysfunction, and atherosclerosis (Constantino-Jonapa et al. 2025; Li et al. 2024). In our Obese-Smoker cohort, the inferred depletion of butyrate synthesis KOs and enrichment of LPS biosynthesis KOs aligns with this pathogenic framework and may help explain the elevated inflammatory markers (CRP) and insulin resistance (HOMA-IR) observed in these individuals. Geographic variation in microbiome composition is well-documented, with factors such as diet, genetics, antibiotic exposure, and lifestyle shaping population-specific microbial signatures (Kumar and Bhadury 2023). A comparative metagenomic study of healthy individuals from Thailand and Norway revealed distinct geographic signatures in antimicrobial resistance gene carriage, demonstrating that local exposures shape microbial functional potential even when taxonomic composition is relatively conserved (Tansirichaiya et al. 2025). While our Egyptian cohort provides valuable data from an understudied region, the consistency of our core findings (e.g., Proteobacteria expansion, pathobiont enrichment) with studies from diverse populations supports their broader relevance (Shin et al. 2015). However, population-specific differences in baseline microbiota suggest that the specific oral biomarker *F0058* (Saccharimonadaceae) identified in our study may require validation in other geographic settings (Ma et al. 2022; Srila et al. 2025).

This study has several limitations. First, the cross-sectional design establishes associations but cannot infer causality between smoking, obesity, and the observed microbial shifts; longitudinal or interventional studies are needed to clarify temporal relationships. Second, while 16 S rRNA sequencing provides robust community profiling, its taxonomic resolution is limited at the species level, and functional insights are inferred rather than directly measured. Future research using shotgun metagenomics and metabolomics could help validate predicted functional changes, such as alterations in butyrate metabolism. Third, although we collected detailed data on smoking intensity (cigarettes/day and pack-years), we did not differentiate between cigarette types (e.g., menthol vs. non-menthol) or include other tobacco products. Future studies should incorporate comprehensive tobacco product characterization to explore potential differential effects on the microbiota. Fourth, regarding oral health status, our study employed stringent exclusion criteria specifically designed to eliminate confounding by active oral pathology, participants with dental caries, oral abscesses, untreated cavitated lesions, periodontal disease, or periodontal pockets ≥ 4 mm were excluded, ensuring all individuals were periodontally healthy. While this design choice intentionally isolated the effects of smoking and obesity independent of pre-existing oral disease, it precluded analysis of correlations with oral health parameters such as DMFT index or gingivitis scores. Fifth, although total energy intake (kcal) was adjusted using FFQ/24-h recall data, we did not explicitly model dietary composition (e.g., fiber quantity/quality, fat quality, and free sugars), which may have contributed to residual confounding. Finally, recruiting participants from a single geographic region may limit generalizability to populations with different genetic and lifestyle backgrounds. Nonetheless, the consistency of our findings with those of the global literature supports their broader relevance, underscoring the need for interventional studies to confirm the causal pathways suggested by our analyses.

## Conclusions

Obesity and smoking synergistically alter the orointestinal microbiome axis through distinct yet interconnected mechanisms: pathobiont expansion, depletion of protective commensals, and smoking-associated covariation across habitats. The oral microbiome is vulnerable and serves as an early indicator of dysbiosis. Compartment‑specific signatures, especially the oral biomarker F0058 and shared taxa such as *Neisseria*, show promising discriminatory performance; external validation is needed to confirm their diagnostic utility. Targeting orointestinal microbial homeostasis represents a promising strategy for mitigating metabolic and inflammatory sequelae in this high-risk population.

## Supplementary Information

Below is the link to the electronic supplementary material.

Supplementary Material 1

Supplementary Material 2

## Data Availability

The raw 16 S rRNA sequences were deposited in the NCBI database with the Sequence Read Archive (SRA) under a bioproject Accession number: PRJNA1018993. R scripts are available at [https://gist.github.com/Mohammedramadan2012](https:/gist.github.com/Mohammedramadan2012) .

## References

[CR3] Al Bataineh MT, Dash NR, Elkhazendar M, Alnusairat DMH, Darwish IMI, Al-Hajjaj MS, Hamid Q (2020) Revealing oral microbiota composition and functionality associated with heavy cigarette smoking. J Transl Med 18(1):421. 10.1186/s12967-020-02579-3 10.1186/s12967-020-02579-3 PMC7653996 33167991

[CR1] Al-Marzooq F, Al Kawas S, Rahman B, Shearston JA, Saad H, Benzina D, Weitzman M (2022) Supragingival microbiome alternations as a consequence of smoking different tobacco types and its relation to dental caries. Sci Rep 12(1):2861. 10.1038/s41598-022-06907-z 35190583 10.1038/s41598-022-06907-z PMC8861055

[CR2] Al-Zyoud W, Hajjo R, Abu-Siniyeh A, Hajjaj S (2020) Salivary microbiome and cigarette smoking: a first of its kind investigation in Jordan. Int J Environ Res Public Health 17(1):256. 10.3390/ijerph17010256 10.3390/ijerph17010256 PMC6982339 31905907

[CR4] Anderson MJ (2001) A new method for non-parametric multivariate analysis of variance. Austral Ecol 26(1):32–46. 10.1111/j.1442-9993.2001.01070.pp.x

[CR5] Aoun A, Darwish F, Hamod N (2020) The influence of the gut microbiome on obesity in adults and the role of probiotics, prebiotics, and synbiotics for weight loss. Prev Nutr food Sci 25(2):113–123. 10.3746/pnf.2020.25.2.113 32676461 10.3746/pnf.2020.25.2.113 PMC7333005

[CR6] Arumugam M, Raes J, Pelletier E, Le Paslier D, Yamada T, Mende DR, Fernandes GR, Tap J, Bruls T, Batto J-M, Bertalan M, Borruel N, Casellas F, Fernandez L, Gautier L, Hansen T, Hattori M, Hayashi T, Kleerebezem M, Kurokawa K, Leclerc M, Levenez F, Manichanh C, Nielsen HB, Nielsen T, Pons N, Poulain J, Qin J, Sicheritz-Ponten T, Tims S, Torrents D, Ugarte E, Zoetendal EG, Wang J, Guarner F, Pedersen O, de Vos WM, Brunak S, Doré J, Antolín M, Artiguenave F, Blottiere HM, Almeida M, Brechot C, Cara C, Chervaux C, Cultrone A, Delorme C, Denariaz G, Dervyn R, Foerstner KU, Friss C, van de Guchte M, Guedon E, Haimet F, Huber W, van Hylckama-Vlieg J, Jamet A, Juste C, Kaci G, Knol J, Lakhdari O, Layec S, Le Roux K, Maguin E, Mérieux A, Melo Minardi R, M’Rini C, Muller J, Oozeer R, Parkhill J, Renault P, Rescigno M, Sanchez N, Sunagawa S, Torrejon A, Turner K, Vandemeulebrouck G, Varela E, Winogradsky Y, Zeller G, Weissenbach J, Ehrlich SD, Bork P (2011) Enterotypes of the human gut microbiome. Nature 473(7346):174–180. 10.1038/nature09944 21508958 10.1038/nature09944 PMC3728647

[CR7] Arweiler NB, Netuschil L (2016) The oral microbiota. Adv Exp Med Biol 902:45–60. 10.1007/978-3-319-31248-4_4 27161350 10.1007/978-3-319-31248-4_4

[CR8] Aßhauer KP, Wemheuer B, Daniel R, Meinicke P (2015) Tax4Fun: predicting functional profiles from metagenomic 16S rRNA data. Bioinformatics 31(17):2882–2884. 10.1093/bioinformatics/btv287 25957349 10.1093/bioinformatics/btv287 PMC4547618

[CR9] Baima G, Ribaldone DG, Romano F, Aimetti M, Romandini M (2023) The gum-gut axis: periodontitis and the risk of gastrointestinal cancers. Cancers. 10.3390/cancers15184594 10.3390/cancers15184594 PMC10526746 37760563

[CR10] Batatinha HAP, Rosa Neto JC, Krüger KJEIR (2019) Inflammatory features of obesity and smoke exposure and the immunologic effects of exercise. Exerc Immunol Rev 25:96–111 30753132

[CR11] Benjamini Y, Hochberg Y (1995) Controlling the false discovery rate: a practical and powerful approach to multiple testing. J R Stat Soc: Ser B (Methodol) 57(1):289–300. 10.1111/j.2517-6161.1995.tb02031.x

[CR12] Beverly ML-S, Chaudhary PP, Dabdoub SM, Kim S, Chatzakis E, Williamson K, Ganesan SM, Yadav M, Ratley G, D’Souza BN, Myles IA, Kumar PS (2025) Toxic cultures: e-cigarettes and the oral microbial exposome. npj Biofilms Microbiomes 11(1):66. 10.1038/s41522-025-00709-7 40280980 10.1038/s41522-025-00709-7 PMC12032151

[CR13] Bombin A, Yan S, Bombin S, Mosley JD, Ferguson JF (2022) Obesity influences composition of salivary and fecal microbiota and impacts the interactions between bacterial taxa. Physiol Rep 10(7):e15254. 10.14814/phy2.15254 10.14814/phy2.15254 PMC8980904 35384379

[CR14] Calderaro J, Ziol M, Paradis V, Zucman-Rossi J (2019) Molecular and histological correlations in liver cancer. J Hepatol 71(3):616–630. 10.1016/j.jhep.2019.06.001 31195064 10.1016/j.jhep.2019.06.001

[CR15] Caliri AW, Tommasi S, Besaratinia A (2021) Relationships among smoking, oxidative stress, inflammation, macromolecular damage, and cancer. Mutat Research/Reviews Mutat Res 787:108365. 10.1016/j.mrrev.2021.108365 10.1016/j.mrrev.2021.108365 PMC8287787 34083039

[CR16] Callahan BJ, McMurdie PJ, Rosen MJ, Han AW, Johnson AJA, Holmes SP (2016) DADA2: High-resolution sample inference from Illumina amplicon data. Nat Methods 13(7):581–583. 10.1038/nmeth.3869 27214047 10.1038/nmeth.3869 PMC4927377

[CR17] Cao C, Lai T, Li M, Zhou H, Lv D, Deng Z, Ying S, Chen Z, Li W, Shen H (2016) Smoking-promoted oxidative DNA damage response is highly correlated to lung carcinogenesis. Oncotarget 7(14):18919–18926. 10.18632/oncotarget.7810 26942876 10.18632/oncotarget.7810 PMC4951340

[CR18] Chai RC, Lim Y, Frazer IH, Wan Y, Perry C, Jones L, Lambie D, Punyadeera C (2016) A pilot study to compare the detection of HPV-16 biomarkers in salivary oral rinses with tumour p16INK4a expression in head and neck squamous cell carcinoma patients. BMC Cancer 16(1):178. 10.1186/s12885-016-2217-1 26940728 10.1186/s12885-016-2217-1 PMC4778285

[CR19] Chattopadhyay S, Malayil L, Chopyk J, Smyth E, Kulkarni P, Raspanti G, Thomas SB, Sapkota A, Mongodin EF, Sapkota AR (2024) Oral microbiome dysbiosis among cigarette smokers and smokeless tobacco users compared to non-users. Sci Rep 14(1):10394. 10.1038/s41598-024-60730-2 38710815 10.1038/s41598-024-60730-2 PMC11074290

[CR20] Chen B, Sun L, Zeng G, Shen Z, Wang K, Yin L, Xu F, Wang P, Ding Y, Nie Q, Wu Q, Zhang Z, Xia J, Lin J, Luo Y, Cai J, Krausz KW, Zheng R, Xue Y, Zheng M-H, Li Y, Yu C, Gonzalez FJ, Jiang C (2022) Gut bacteria alleviate smoking-related NASH by degrading gut nicotine. Nature 610(7932):562–568. 10.1038/s41586-022-05299-4 36261549 10.1038/s41586-022-05299-4 PMC9589931

[CR21] Chopyk DM, Grakoui A (2020) Contribution of the intestinal microbiome and gut barrier to hepatic disorders. Gastroenterology 159(3):849–863. 10.1053/j.gastro.2020.04.077 32569766 10.1053/j.gastro.2020.04.077 PMC7502510

[CR22] Constantino-Jonapa LA, Aguilar-Villegas OR, Hernández-Ruiz P, Escalona-Montaño AR, Pallecchi M, González-Pacheco H, Bartolucci G, Baldi S, Amezcua-Guerra LM, Amedei A, Aguirre-García MM (2025) The link between inflammatory/ SCFA profiles and oral/gut microbiome: an observational study in patients with ST-segment elevation myocardial infarction. Curr Res Microb Sci 9:100423. 10.1016/j.crmicr.2025.100423 40612273 10.1016/j.crmicr.2025.100423 PMC12221840

[CR23] Cryan JF, O’Riordan KJ, Cowan CSM, Sandhu KV, Bastiaanssen TFS, Boehme M, Codagnone MG, Cussotto S, Fulling C, Golubeva AV, Guzzetta KE, Jaggar M, Long-Smith CM, Lyte JM, Martin JA, Molinero-Perez A, Moloney G, Morelli E, Morillas E, O’Connor R, Cruz-Pereira JS, Peterson VL, Rea K, Ritz NL, Sherwin E, Spichak S, Teichman EM, van de Wouw M, Ventura-Silva AP, Wallace-Fitzsimons SE, Hyland N, Clarke G, Dinan TG (2019) The microbiota-gut-brain axis. Physiol Rev 99(4):1877–2013. 10.1152/physrev.00018.2018 31460832 10.1152/physrev.00018.2018

[CR24] Csardi MG (2013) Package ‘igraph’. Last accessed 3(09):2013

[CR25] Deo PN, Deshmukh R (2019) Oral microbiome: unveiling the fundamentals. J Oral Maxillofac Pathol: JOMFP 23(1):122–128. 10.4103/jomfp.JOMFP_304_18 31110428 10.4103/jomfp.JOMFP_304_18 PMC6503789

[CR26] Descamps P, Dixon S, Bosch Jose FX, Kyrgiou M, Monsonego J, Neisingh O, Nguyen L, O’Connor M, Smith JS (2024) Turning the tide—recommendations to increase cervical cancer screening among women who are underscreened. 166(S1):3–21. 10.1002/ijgo.15600 10.1002/ijgo.15600 38853590

[CR27] Dhariwal A, Chong J, Habib S, King IL, Agellon LB, Xia J (2017) MicrobiomeAnalyst: a web-based tool for comprehensive statistical, visual and meta-analysis of microbiome data. Nucleic Acids Res 45(W1):W180–w188. 10.1093/nar/gkx295 28449106 10.1093/nar/gkx295 PMC5570177

[CR28] Duan Y, Xu C, Wang W, Wang X, Xu N, Zhong J, Gong W, Zheng W, Wu Y-H, Myers A, Chu L, Lu Y, Delzell E, Hsing AW, Yu M, He W, Zhu S (2025) Smoking-related gut microbiota alteration is associated with obesity and obesity-related diseases: results from two cohorts with sibling comparison analyses. BMC Med 23(1):146. 10.1186/s12916-025-03969-4 40059170 10.1186/s12916-025-03969-4 PMC11892230

[CR29] El Menofy NG, Ramadan M, Abdelbary ER, Ibrahim HG, Azzam AI, Ghit MM, Ezz AS, Gazar YA, Salah M (2022) Bacterial compositional shifts of gut microbiomes in patients with rheumatoid arthritis in association with disease activity. Microorganisms. 10.3390/microorganisms10091820 10.3390/microorganisms10091820 PMC9505928 36144422

[CR30] Eva J, Olsson T, Alfredsson L, Hedström AK (2025) Smoking and obesity interact to adversely affect disease progression and cognitive performance in multiple sclerosis. Eur J Neurol 32(2):e70058. 10.1111/ene.70058 39905709 10.1111/ene.70058 PMC11794246

[CR31] Fan Y, Pedersen O (2021) Gut microbiota in human metabolic health and disease. Nat Rev Microbiol 19(1):55–71. 10.1038/s41579-020-0433-9 32887946 10.1038/s41579-020-0433-9

[CR32] Fernandes R, Viana SD, Nunes S, Reis F (2019) Diabetic gut microbiota dysbiosis as an inflammaging and immunosenescence condition that fosters progression of retinopathy and nephropathy. Biochim Biophys Acta (BBA) Mol Basis Dis 1865(7):1876–1897. 10.1016/j.bbadis.2018.09.032 10.1016/j.bbadis.2018.09.032 30287404

[CR33] Formisano A, Russo MD, Russo P, Siani A, Hinojosa-Nogueira D, Navajas-Porras B, Toledano-Marín Á, Pastoriza S, Blasco T, Lerma-Aguilera A, Francino MP, Planes FJ, González-Vigil V, Rufián-Henares JÁ, Lauria F (2024) Development and validation of a self-administered semiquantitative food frequency questionnaire focused on gut microbiota: the Stance4Health-FFQ. Nutrients 16:4064 10.3390/nu16234064 PMC11643877 39683458

[CR34] Foster E, Bradley J (2018) Methodological considerations and future insights for 24-hour dietary recall assessment in children. Nutr Res 51:1–11. 10.1016/j.nutres.2017.11.001 29673539 10.1016/j.nutres.2017.11.001

[CR35] Goldfarb DM, Tilley P, Al-Rawahi GN, Srigley JA, Ford G, Pedersen H, Pabbi A, Hannam-Clark S, Charles M, Dittrick M, Gadkar VJ, Pernica JM, Hoang LMN (2021) Self-collected saline gargle samples as an alternative to health care worker-collected nasopharyngeal swabs for COVID-19 diagnosis in outpatients. J Clin Microbiol. 10.1128/jcm.02427-20 10.1128/JCM.02427-20 PMC8092743 33514627

[CR36] Han J, Zhang B, Zhang Y, Yin T, Cui Y, Liu J, Yang Y, Song H, Shang D (2023) Gut microbiome: decision-makers in the microenvironment of colorectal cancer. Front Cell Infect Microbiol 13:1299977. 10.3389/fcimb.2023.1299977 38156313 10.3389/fcimb.2023.1299977 PMC10754537

[CR37] Hills RD Jr., Pontefract BA, Mishcon HR, Black CA, Sutton SC, Theberge CR (2019) Gut microbiome: profound implications for diet and disease. Nutrients. 10.3390/nu11071613 10.3390/nu11071613 PMC6682904 31315227

[CR38] Huang C, Shi G (2019) Smoking and microbiome in oral, airway, gut and some systemic diseases. J translational Med 17(1):225. 10.1186/s12967-019-1971-7 10.1186/s12967-019-1971-7 PMC6632217 31307469

[CR39] Huwart SJP, Fayt C, Gangarossa G, Luquet S, Cani PD, Everard A (2024) TLR4-dependent neuroinflammation mediates LPS-driven food-reward alterations during high-fat exposure. J Neuroinflamm 21(1):305. 10.1186/s12974-024-03297-z 10.1186/s12974-024-03297-z PMC11585241 39580436

[CR40] Imade EE, Obayagbona NO (2024a) Impact of cigarette smoking on gut microbial dysbiosis: a structured literature review 10.1017/gmb.2024.3 PMC11658948 39703543

[CR41] Imade EE, Obayagbona NO (2024b) Impact of cigarette smoking on gut microbial dysbiosis: a structured literature review. Gut Microbiome 5:e13. 10.1017/gmb.2024.3 39703543 10.1017/gmb.2024.3 PMC11658948

[CR42] Jones J, Reinke SN, Ali A, Palmer DJ, Christophersen CT (2021) Fecal sample collection methods and time of day impact microbiome composition and short chain fatty acid concentrations. Sci Rep 11(1):13964. 10.1038/s41598-021-93031-z 34234185 10.1038/s41598-021-93031-z PMC8263620

[CR43] Kanehisa M, Goto S (2000) KEGG: kyoto encyclopedia of genes and genomes. Nucleic Acids Res 28(1):27–30. 10.1093/nar/28.1.27 10592173 10.1093/nar/28.1.27 PMC102409

[CR44] Kato I, Nechvatal JM, Dzinic S, Basson MD, Majumdar AP, Ram JL (2010) Smoking and other personal characteristics as potential predictors for fecal bacteria populations in humans. Med Sci monitor: Int Med J experimental Clin Res 16(1):Cr1–7 PMC4929991 20037488

[CR45] Kelly BJ, Gross R, Bittinger K, Sherrill-Mix S, Lewis JD, Collman RG, Bushman FD (2015) Power and sample-size estimation for microbiome studies using pairwise distances and PERMANOVA. Bioinformatics 31(15):2461–2468. 10.1093/bioinformatics/btv183 25819674 10.1093/bioinformatics/btv183 PMC4514928

[CR46] Kim M-H, Yun KE, Kim J, Park E, Chang Y, Ryu S, Kim H-L, Kim H-N (2020) Gut microbiota and metabolic health among overweight and obese individuals. Sci Rep 10(1):19417. 10.1038/s41598-020-76474-8 33173145 10.1038/s41598-020-76474-8 PMC7655835

[CR47] Knights D, Costello EK, Knight R (2011) Supervised classification of human microbiota. FEMS Microbiol Rev 35(2):343–359. 10.1111/j.1574-6976.2010.00251.x 21039646 10.1111/j.1574-6976.2010.00251.x

[CR48] Kong Y, Yang H, Nie R, Zhang X, Zuo F, Zhang H, Nian X (2025) Obesity: pathophysiology and therapeutic interventions. Mol Biomed 6(1):25. 10.1186/s43556-025-00264-9 40278960 10.1186/s43556-025-00264-9 PMC12031720

[CR49] Kumar G, Bhadury P (2023) Exploring the influences of geographical variation on sequence signatures in the human gut microbiome. J Genet 102(2):51. 10.1007/s12041-023-01448-4 38073168

[CR50] Kumar R, Shukla SK, Gupta A, Vibhuti A (2025) Unveiling the gut microbiome’s role in obesity and insulin resistance. In: Nandave M, Pati Pandey R, Upadhyay J (eds) Gut health and metabolic syndrome: optimizations with microbiome innovations. Springer, Singapore, pp 145–167

[CR51] Kurtz ZD, Müller CL, Miraldi ER, Littman DR, Blaser MJ, Bonneau RA (2015) Sparse and compositionally robust inference of microbial ecological networks. PLoS Comput Biol 11(5):e1004226. 10.1371/journal.pcbi.1004226 25950956 10.1371/journal.pcbi.1004226 PMC4423992

[CR52] Leite G, Barlow GM, Hosseini A, Parodi G, Pimentel ML, Wang J, Fiorentino A, Rezaie A, Pimentel M, Mathur R (2022) Smoking has disruptive effects on the small bowel luminal microbiome. Sci Rep 12(1):6231. 10.1038/s41598-022-10132-z 35422064 10.1038/s41598-022-10132-z PMC9010470

[CR53] Li X, Li Q, Wang L, Ding H, Wang Y, Liu Y, Gong T (2024) The interaction between oral microbiota and gut microbiota in atherosclerosis. Front Cardiovasc Med 11:1406220 10.3389/fcvm.2024.1406220 PMC11199871 38932989

[CR54] Lloyd-Price J, Abu-Ali G, Huttenhower C (2016) The healthy human microbiome. Genome Med 8:51. 10.1186/s13073-016-0307-y 27122046 10.1186/s13073-016-0307-y PMC4848870

[CR55] López-Tenorio II, Constantino-Jonapa LA, Jaimez-Alvarado S, Reyes-Martínez S, Escalona-Montaño AR, Tavera-Alonso C, Valdez-Gómez R, Menicatti M, Bartolucci G, Niccolai E, Simone B, Amedei A, Ávila-Vanzzini N, Aguirre-García MM (2026) Age-related diversity of the oral and gut microbiome and its correlation with systemic fatty acids and cytokine profiles in healthy subjects. Exp Gerontol 215:113046. 10.1016/j.exger.2026.113046 41592670 10.1016/j.exger.2026.113046

[CR56] Love MI, Huber W, Anders S (2014) Moderated estimation of fold change and dispersion for RNA-seq data with DESeq2. Genome Biol 15(12):550. 10.1186/s13059-014-0550-8 25516281 10.1186/s13059-014-0550-8 PMC4302049

[CR57] Lu H, Zou P, Zhang Y, Zhang Q, Chen Z, Chen F (2022) The sampling strategy of oral microbiome. iMeta 1(2):e23. 10.1002/imt2.23 38868567 10.1002/imt2.23 PMC10989882

[CR58] Ma G, Qiao Y, Shi H, Zhou J, Li Y (2022) Comparison of the oral microbiota structure among people from the same ethnic group living in different environments. Biomed Res Int 2022:6544497. 10.1155/2022/6544497 35800217 10.1155/2022/6544497 PMC9256442

[CR59] Maki KA, Ganesan SM, Meeks B, Farmer N, Kazmi N, Barb JJ, Joseph PV, Wallen GR (2022) The role of the oral microbiome in smoking-related cardiovascular risk: a review of the literature exploring mechanisms and pathways. J Transl Med 20(1):584. 10.1186/s12967-022-03785-x 10.1186/s12967-022-03785-x PMC9743777 36503487

[CR60] Mehrotra V, Devi P, Bhovi TV, Jyoti B (2010) Mouth as a mirror of systemic diseases. Gomal J Med Sci 8(2)

[CR61] Mei Z, Li D (2022) The role of probiotics in vaginal health. Front Cell Infect Microbiol 12:963868 10.3389/fcimb.2022.963868 10.3389/fcimb.2022.963868 PMC9366906 35967876

[CR62] Mohamed Lotfy AE-W, Hadad Hemeda M, Ezzat Abd El-Aziz Ali A, Metwally Bauomy I, Gamil Hammed Shola M (2020) Prevalence of pre diabetes and diabetes mellitus among Al-Azhar University male students Hostel in Cairo Egypt Cross section study. Al-Azhar Med J 49(3):931–938. 10.21608/amj.2020.91617

[CR63] Mohamed MNA, El-Hamd Mohamed A-ES (2021) Study of Health Behaviours and Lifestyle Characterstics among Medical Students at Al-Azhar University, Assuit Branch. Med J Cairo Univ 89(December):2555–2569. 10.21608/mjcu.2021.217396

[CR65] Mohammed S, Hosny MF, Mohamed W, Ezz Eldeen ME, Badary MS (2022) The relationship between the gut microbiota shifts and the inflammatory biomarkers in obese and normal weight adults. Microbes Infect Dis 3(3):703–713. 10.21608/mid.2022.113960.1223

[CR64] Mohammed LI, Razali R, Zakaria ZZ, Benslimane FM, Cyprian F, Al-Asmakh M (2024) Smoking induced salivary microbiome dysbiosis and is correlated with lipid biomarkers. BMC Oral Health 24(1):608. 10.1186/s12903-024-04340-4 38796419 10.1186/s12903-024-04340-4 PMC11127352

[CR66] Muluke M, Gold T, Kiefhaber K, Al-Sahli A, Celenti R, Jiang H, Cremers S, Van Dyke T, Schulze-Späte U (2016) Diet-induced obesity and its differential impact on periodontal bone loss. J Dent Res 95(2):223–229. 10.1177/0022034515609882 26450512 10.1177/0022034515609882 PMC4720954

[CR67] Neri-Rosario D, Martínez-López YE, Esquivel-Hernández DA, Sánchez-Castañeda JP, Padron-Manrique C, Vázquez-Jiménez A, Giron-Villalobos D, Resendis-Antonio O (2023) Dysbiosis signatures of gut microbiota and the progression of type 2 diabetes: a machine learning approach in a Mexican cohort. Front Endocrinol 14:1170459. 10.3389/fendo.2023.1170459 10.3389/fendo.2023.1170459 PMC10333697 37441494

[CR68] Ogunrinola GA, Oyewale JO, Oshamika OO, Olasehinde GI (2020) The human microbiome and its impacts on health. Int J Microbiol 2020:8045646. 10.1155/2020/8045646 32612660 10.1155/2020/8045646 PMC7306068

[CR69] Park SY, Hwang BO, Lim M, Ok SH, Lee SK, Chun KS, Park KK, Hu Y, Chung WY, Song NY (2021) Oral-gut microbiome axis in gastrointestinal disease and cancer. Cancers. 10.3390/cancers13092124 10.3390/cancers13092124 PMC8125773 33924899

[CR70] Pascal V, Pozuelo M, Borruel N, Casellas F, Campos D, Santiago A, Martinez X, Varela E, Sarrabayrouse G, Machiels K, Vermeire S, Sokol H, Guarner F, Manichanh C (2017) A microbial signature for Crohn’s disease. Gut 66(5):813. 10.1136/gutjnl-2016-313235 28179361 10.1136/gutjnl-2016-313235 PMC5531220

[CR71] Pasolli E, Truong DT, Malik F, Waldron L, Segata N (2016) Machine learning meta-analysis of large metagenomic datasets: tools and biological insights. PLoS Comput Biol 12(7):e1004977. 10.1371/journal.pcbi.1004977 27400279 10.1371/journal.pcbi.1004977 PMC4939962

[CR72] Peng X, Cheng L, You Y, Tang C, Ren B, Li Y, Xu X, Zhou X (2022) Oral microbiota in human systematic diseases. Int J Oral Sci 14(1):14. 10.1038/s41368-022-00163-7 35236828 10.1038/s41368-022-00163-7 PMC8891310

[CR73] Petakh P, Oksenych V, Kamyshnyi A (2023) The F/B ratio as a biomarker for inflammation in COVID-19 and T2D: impact of metformin. Biomed Pharmacother 163:114892. 10.1016/j.biopha.2023.114892 37196542 10.1016/j.biopha.2023.114892 PMC10183625

[CR74] Pirih FQ, Monajemzadeh S, Singh N, Sinacola RS, Shin JM, Chen T, Fenno JC, Kamarajan P, Rickard AH, Travan S, Paster BJ, Kapila Y (2021) Association between metabolic syndrome and periodontitis: the role of lipids, inflammatory cytokines, altered host response, and the microbiome. Periodontol 2000 87(1):50–75. 10.1111/prd.12379 34463996 10.1111/prd.12379 PMC8457155

[CR75] Pleasants RA, Rivera MP, Tilley SL, Bhatt SP (2020) Both duration and pack-years of tobacco smoking should be used for clinical practice and research. Annals Am Thorac Soc 17(7):804–806. 10.1513/AnnalsATS.202002-133VP 10.1513/AnnalsATS.202002-133VP PMC7405110 32348693

[CR76] Qiu Z, Chen X, Geng T, Wan Z, Lu Q, Li L, Zhu K, Zhang X, Liu Y, Lin X, Chen L, Shan Z, Liu L, Pan A, Liu G (2022) Associations of serum carotenoids with risk of cardiovascular mortality among individuals with type 2 diabetes: results from NHANES. Diabetes Care 45(6):1453–1461. 10.2337/dc21-2371 35503926 10.2337/dc21-2371

[CR77] Quast C, Pruesse E, Yilmaz P, Gerken J, Schweer T, Yarza P, Peplies J, Glöckner FO (2012) The SILVA ribosomal RNA gene database project: improved data processing and web-based tools. Nucleic Acids Res 41D1:D590–D596. 10.1093/nar/gks1219 10.1093/nar/gks1219 PMC3531112 23193283

[CR78] Queen J, Cing Z, Minsky H, Nandi A, Southward T, Ferri J, McMann M, Iyadorai T, Vadivelu J, Roslani A, Loke MF, Wanyiri J, White JR, Drewes JL, Sears CL (2025) Fusobacterium nucleatum is enriched in invasive biofilms in colorectal cancer. npj Biofilms Microbiomes 11(1):81. 10.1038/s41522-025-00717-7 40394001 10.1038/s41522-025-00717-7 PMC12092649

[CR79] R Core Team R (2021) R: A language and environment for statistical. R Foundation for Statistical Computing, Vienna, Austria

[CR80] Rajasekaran JJ, Krishnamurthy HK, Bosco J, Jayaraman V, Krishna K, Wang T, Bei K (2024) Oral microbiome: a review of its impact on oral and systemic health. Microorganisms. 10.3390/microorganisms12091797 10.3390/microorganisms12091797 PMC11434369 39338471

[CR81] Ruan Q, Guan P, Qi W, Li J, Xi M, Xiao L, Zhong S, Ma D, Ni J (2023) Porphyromonas gingivalis regulates atherosclerosis through an immune pathway. Front Immunol 14 . 10.3389/fimmu.2023.1103592 10.3389/fimmu.2023.1103592 PMC10043234 36999040

[CR82] Saad SAEF, Abu-Elnaga NA, Ghany ABA, Hassan AFA (2021) The impact of smoking on semen quality and ICSI outcome in the obese men. Egypt J Hosp Med 84(1):2256–2261 .10.21608/ejhm.2021.181219

[CR83] Sarafidou K, Alexakou E, Talioti E, Bakopoulou A, Anastassiadou V (2024) The oral microbiome in older adults—a state-of-the-art review. Arch Gerontol Geriatr Plus 1(4):100061. 10.1016/j.aggp.2024.100061

[CR84] Seekatz AM, Safdar N, Khanna S (2022) The role of the gut microbiome in colonization resistance and recurrent Clostridioides difficile infection. Therapeutic Adv Gastroenterol 15:17562848221134396. 10.1177/17562848221134396 10.1177/17562848221134396 PMC9679343 36425405

[CR85] Segata N, Izard J, Waldron L, Gevers D, Miropolsky L, Garrett WS, Huttenhower C (2011) Metagenomic biomarker discovery and explanation. Genome Biol 12(6):R60. 10.1186/gb-2011-12-6-r60 21702898 10.1186/gb-2011-12-6-r60 PMC3218848

[CR86] Shabayek S, Abdellah AM, Salah M, Ramadan M, Fahmy N (2022) Alterations of the vaginal microbiome in healthy pregnant women positive for group B Streptococcus colonization during the third trimester. BMC Microbiol 22(1):313. 10.1186/s12866-022-02730-8 36544085 10.1186/s12866-022-02730-8 PMC9769055

[CR87] Shaffer M, Lozupone C (2018) Prevalence and source of fecal and oral bacteria on infant, child, and adult hands. mSystems 3(1):e00192–e00117. 10.1128/mSystems.00192-17 29359197 10.1128/mSystems.00192-17 PMC5768791

[CR88] Shaheen N, Shaheen A, Diab RA, Saad AM, Abdelwahab OA, Soliman S, Hefnawy MT, Ramadan A, Meshref M, Nashwan AJ (2023) Association of serum leptin and ghrelin levels with smoking status on body weight: a systematic review and meta-analysis. Front Psychiatry 14 . 10.3389/fpsyt.2023.1296764 10.3389/fpsyt.2023.1296764 PMC10725976 38111614

[CR89] Shapiro H, Goldenberg K, Ratiner K, Elinav E (2022) Smoking-induced microbial dysbiosis in health and disease. Clin Sci 136(18):1371–1387. 10.1042/CS20220175 10.1042/CS20220175 PMC9527826 36156126

[CR90] Shin N-R, Whon TW, Bae J-W (2015) ) Proteobacteria: microbial signature of dysbiosis in gut microbiota. Trends Biotechnol 33(9):496–503. 10.1016/j.tibtech.2015.06.011 26210164 10.1016/j.tibtech.2015.06.011

[CR91] Shoji M, Sasaki Y, Abe Y, Nishise S, Yaoita T, Yagi M, Mizumoto N, Kon T, Onozato Y, Sakai TJJO (2021) Characteristics of the gut microbiome profile in obese patients with colorectal cancer. JGH Open 5(4):498–507. 10.1002/jgh3.12529 33860101 10.1002/jgh3.12529 PMC8035457

[CR92] Singh V, Lee G, Son H, Koh H, Kim ES, Unno T, Shin JH (2022) Butyrate producers, The sentinel of gut: their intestinal significance with and beyond butyrate, and prospective use as microbial therapeutics. Front Microbiol 13:1103836. 10.3389/fmicb.2022.1103836 36713166 10.3389/fmicb.2022.1103836 PMC9877435

[CR93] Sreevatshan KS, Nair VG, Srinandan CS, Malli Mohan GB (2022) Tools to study gut microbiome. In: Tripathi AK, Kotak M (eds) Gut microbiome in neurological health and disorders. Springer, Singapore, pp 253–270 . 10.1007/978-981-19-4530-4_15

[CR94] Srila W, Sripilai K, Binlateh T, Thammanichanon P, Tiskratok W, Noisa P, Jitprasertwong P (2025) Relationship between the salivary microbiome and oral malodor metabolites in older thai individuals with periodontitis and the cytotoxic effects of malodor compounds on human oral squamous carcinoma (HSC-4) cells. Dent J 13:36 . https://www.mdpi.com/2304-6767/13/1/36. 10.3390/dj13010036 PMC11764442 39851614

[CR95] Sublette MG, Cross T-WL, Korcarz CE, Hansen KM, Murga-Garrido SM, Hazen SL, Wang Z, Oguss MK, Rey FE, Stein JH (2020) Effects of smoking and smoking cessation on the intestinal microbiota. J Clin Med 9(9):2963. https://www.mdpi.com/2077-0383/9/9/2963. 32937839 10.3390/jcm9092963 PMC7564179

[CR96] Suzuki N, Nakano Y, Yoneda M, Hirofuji T, Hanioka T (2022) The effects of cigarette smoking on the salivary and tongue microbiome. Clin Exp Dent Res 8(1):449–456. 10.1002/cre2.489 10.1002/cre2.489 PMC8874080 34505401

[CR97] Tansirichaiya S, Songsomboon K, Wigand J, Winje E, Chaianant N, Leartsiwawinyu W, Al-Haroni M (2025) Geographic signatures in the oral resistome: a comparative metagenomic analysis of healthy individuals from Thailand and Norway. J Oral Microbiol 17(1):2589656. 10.1080/20002297.2025.2589656 41367410 10.1080/20002297.2025.2589656 PMC12683769

[CR98] Tapsell LC, Neale EP, Satija A, Hu FB (2016) Foods, nutrients, and dietary patterns: interconnections and implications for dietary guidelines. Adv Nutr 7(3):445–454. 10.3945/an.115.011718 27184272 10.3945/an.115.011718 PMC4863273

[CR99] Tawfik SA, Azab M, Ramadan M, Shabayek S, Abdellah A, Al Thagfan SS, Salah M (2023) The eradication of helicobacter pylori was significantly associated with compositional patterns of orointestinal axis microbiota. Pathogens 12(6):832 37375522 10.3390/pathogens12060832 PMC10303999

[CR100] Upadhyay P, Wu C-W, Pham A, Zeki AA, Royer CM, Kodavanti UP, Takeuchi M, Bayram H, Pinkerton KE (2023) Animal models and mechanisms of tobacco smoke-induced chronic obstructive pulmonary disease (COPD). J Toxicol Environ Health Part B 26(5):275–305. 10.1080/10937404.2023.2208886 10.1080/10937404.2023.2208886 PMC10718174 37183431

[CR101] van Deuren T, Blaak EE, Canfora EE (2022) Butyrate to combat obesity and obesity-associated metabolic disorders: current status and future implications for therapeutic use. Obes Rev: Off J Int Assoc Study Obes 23(10):e13498. 10.1111/obr.13498 10.1111/obr.13498 PMC9541926 35856338

[CR102] van Dijk MC, Petersen JF, Raber-Durlacher JE, Epstein JB, Laheij A (2024) Diversity and compositional differences in the oral microbiome of oral squamous cell carcinoma patients and healthy controls: a scoping review. Front oral health 5:1366153. 10.3389/froh.2024.1366153 38919733 10.3389/froh.2024.1366153 PMC11196763

[CR103] Vasana J, Niwed K, Kongkiat K, Eleni G, Siam P (2018) Comparison of gut microbiota between lean and obese adult thai individuals. Microbiol Biotechnol Lett 46(3):277–287 10.4014/mbl.1711.11003

[CR104] Verhaar BJH, Prodan A, Nieuwdorp M, Muller M (2020) Gut microbiota in hypertension and atherosclerosis: a review. Nutrients 12(10):2982.https://www.mdpi.com/2072-6643/12/10/2982. 10.3390/nu12102982 PMC7601560 33003455

[CR106] Wang Q, Garrity GM, Tiedje JM, Cole JR (2007) Naïve Bayesian classifier for rapid assignment of rRNA sequences into the new bacterial taxonomy. Appl Environ Microbiol 73(16):5261–5267. 10.1128/AEM.00062-07 17586664 10.1128/AEM.00062-07 PMC1950982

[CR105] Wang P, Dong Y, Jiao J, Zuo K, Han C, Zhao L, Ding S, Yang X, Chen M, Li J (2021) Cigarette smoking status alters dysbiotic gut microbes in hypertensive patients. J Clin Hypertens 23(7):1431–1446. 10.1111/jch.14298 10.1111/jch.14298 PMC8678690 34029428

[CR107] Wirth R, Maróti G, Mihók R, Simon-Fiala D, Antal M, Pap B, Demcsák A, Minarovits J, Kovács KL (2020) A case study of salivary microbiome in smokers and non-smokers in Hungary: analysis by shotgun metagenome sequencing. J Oral Microbiol 12(1):1773067. 10.1080/20002297.2020.1773067 32922678 10.1080/20002297.2020.1773067 PMC7448927

[CR108] Wu J, Peters BA, Dominianni C, Zhang Y, Pei Z, Yang L, Ma Y, Purdue MP, Jacobs EJ, Gapstur SM, Li H, Alekseyenko AV, Hayes RB, Ahn J (2016) Cigarette smoking and the oral microbiome in a large study of American adults. ISME J 10(10):2435–2446. 10.1038/ismej.2016.37 27015003 10.1038/ismej.2016.37 PMC5030690

[CR109] Wu M-C, Nfor ON, Liaw Y-P, Su Y-J, Ho C-C (2025) Associations of cigarette smoking with general and abdominal obesity risks among men in Taiwan. BMC Public Health 25(1):649. 10.1186/s12889-025-21821-5 39962411 10.1186/s12889-025-21821-5 PMC11834593

[CR111] Xiao S, Zhao L (2014) Gut microbiota-based translational biomarkers to prevent metabolic syndrome via nutritional modulation. FEMS Microbiol Ecol 87(2):303–314. 10.1111/1574-6941.12250 24219358 10.1111/1574-6941.12250 PMC4262049

[CR110] Xiao J, Fiscella KA, Gill SR (2020) Oral microbiome: possible harbinger for children’s health. Int J Oral Sci 12(1):12. 10.1038/s41368-020-0082-x 32350240 10.1038/s41368-020-0082-x PMC7190716

[CR112] Yan S, Ma Z, Jiao M, Wang Y, Li A, Ding S (2021) Effects of smoking on inflammatory markers in a healthy population as analyzed via the gut microbiota. Front Cell Infect Microbiol. 10.3389/fcimb.2021.633242 10.3389/fcimb.2021.633242 PMC8342938 34368009

[CR113] Zajmi A, Teo J, Yeo CC (2022) Epidemiology and characteristics of Elizabethkingia spp. infections Southeast Asia. Microorganisms. 10.3390/microorganisms10050882 10.3390/microorganisms10050882 PMC9144721 35630327

[CR114] Zdziarski P, Paściak M, Rogala K, Korzeniowska-Kowal A, Gamian A (2017) Elizabethkingia miricola as an opportunistic oral pathogen associated with superinfectious complications in humoral immunodeficiency: a case report. BMC Infect Dis 17(1):763. 10.1186/s12879-017-2886-7 29233117 10.1186/s12879-017-2886-7 PMC5727958

[CR115] Zeng MY, Inohara N, Nuñez G (2017) Mechanisms of inflammation-driven bacterial dysbiosis in the gut. Mucosal Immunol 10(1):18–26. 10.1038/mi.2016.75 27554295 10.1038/mi.2016.75 PMC5788567

[CR117] Zhang L, Chu J, Hao W, Zhang J, Li H, Yang C, Yang J, Chen X, Wang H (2021) Gut microbiota and type 2 diabetes mellitus: association, mechanism, and translational applications. Mediat Inflamm 2021:5110276. 10.1155/2021/5110276 10.1155/2021/5110276 PMC8384524 34447287

[CR116] Zhang J, Hou L, Lei S, Li Y, Xu G (2025) The causal relationship of cigarette smoking to metabolic disease risk and the possible mediating role of gut microbiota. Ecotoxicol Environ Saf 290:117522. 10.1016/j.ecoenv.2024.117522 39709709 10.1016/j.ecoenv.2024.117522

[CR118] Zhou J, Jiang N, Wang Z, Li L, Zhang J, Ma R, Nie H, Li Z (2017) Influences of pH and iron concentration on the salivary microbiome in individual humans with and without caries. Appl Environ Microbiol. 10.1128/aem.02412-16 10.1128/AEM.02412-16 PMC5288818 27940544

[CR119] Zsálig D, Berta A, Tóth V, Szabó Z, Simon K, Figler M, Pusztafalvi H, Polyák É (2023) A review of the relationship between gut microbiome and obesity. Appl Sci 13:610. 10.3390/app13010610

