# Supplementary material for: Oral-gut microbiome dysbiosis in obese smokers reveals compartment-specific shifts
Source: AMB Express. 2026 Apr 13;16:46. doi: 10.1186/s13568-026-02048-y (PMC13087072; doi:10.1186/s13568-026-02048-y)
Supplement: Supplementary file 2 — Supplementary Material 2 [file 13568_2026_2048_MOESM2_ESM.docx]

**Cross-Sectional 16S rRNA Sequencing Reveals Potential Synergistic Orointestinal Dysbiosis in Obese-Smokers**

**Table S1. Microbiome Stratification by Enterotyping in relation to Obesity and Smoking of Gut Enterotypes**

| **Enterotype** | **Key Genera (Mean RA %)** | **Group Association** | **Significant Features** |
| --- | --- | --- | --- |
| **ET‑G1 (Bacteroides‑driven; Control)** Healthy core | 1. **Bacteroides** (42%) 2. Faecalibacterium (18%) 3. Megasphaera (12%) 4. Collinsella (8%) 5. Ruminococcus_1 (6%) | Controls | • Highest Bacteroides (KW p = 0.006) • Enriched SCFA‑producers • Low methanogen and Proteobacteria signals |
| **ET‑G2 (Prevotella‑driven; Smoker)** Fermentative | 1. **Prevotella 9** (35%) 2. Prevotella 7 (20%) 3. Phascolarctobacterium (10%) 4. Alloprevotella (7%) 5. Dialister (5%) | Smokers | • Elevated Prevotella 9 (KW p = 0.003) • Depleted Bacteroides • Intermediate SCFA and LPS‑producer mix |
| **ET‑G3 (Methanogen/Ruminococcus‑driven; Obese‑Smoker)** Methanogenic | 1. **Ruminococcus_2** (30%) 2. Methanobrevibacter (25%) 3. Eubacterium_copros (15%) 4. Lachnospiraceae_UCG‑008 (8%) 5. Klebsiella (5%) | Obese-Smokers | • Highest methanogen signature (KW p = 0.002) • Loss of core SCFA producers • Expansion of opportunistic Proteobacteria |

**Table S2. Microbiome Stratification by Enterotyping in relation to Obesity and Smoking of Oral Enterotypes**

| **Enterotype** | **Key Genera (Mean RA %)** | **Group Association** | **Significant Features** |
| --- | --- | --- | --- |
| **ET‑O1 (Streptococcus‑Haemophilus; Control)** Homeostatic | 1. **Streptococcus** (38%) 2. Haemophilus (22%) 3. Veillonella (15%) 4. Rothia (8%) 5. Granulicatella (5%) | Controls | • Highest Streptococcus (KW p = 0.008) • Balanced commensals • Low pathobiont burden |
| **ET‑O2 (Veillonella‑Neisseria; Smoker)** Mixed | 1. **Veillonella** (28%) 2. Streptococcus (20%) 3. Neisseria (18%) 4. Prevotella 9 (10%) 5. Alloprevotella (7%) | Smokers | • Elevated Neisseria (KW p = 0.010) • Moderate loss of Elizabethkingia • Increased Proteobacteria |
| **ET‑O3 (Neisseria‑Fusobacterium; Obese‑Smoker)** Pathobiome | 1. **Neisseria** (30%) 2. Fusobacterium (25%) 3. Porphyromonas (12%) 4. Treponema_2 (8%) 5. Campylobacter (5%) | Obese-Smokers | • Highest pathobiont load (KW p = 0.005) • Depleted commensals (Elizabethkingia) • Inflammatory signature |

**Figure S1. Bacterial diversity analysis: Alpha diversity**

Rarefaction curves demonstrate observed amplicon sequence variants (ASVs) across sequencing depths for (A) Oral microbiomes (B) Gut microbiomes. Curves represent individual samples. The rarefaction depth of 26,078 reads was used for downstream analyses.

**
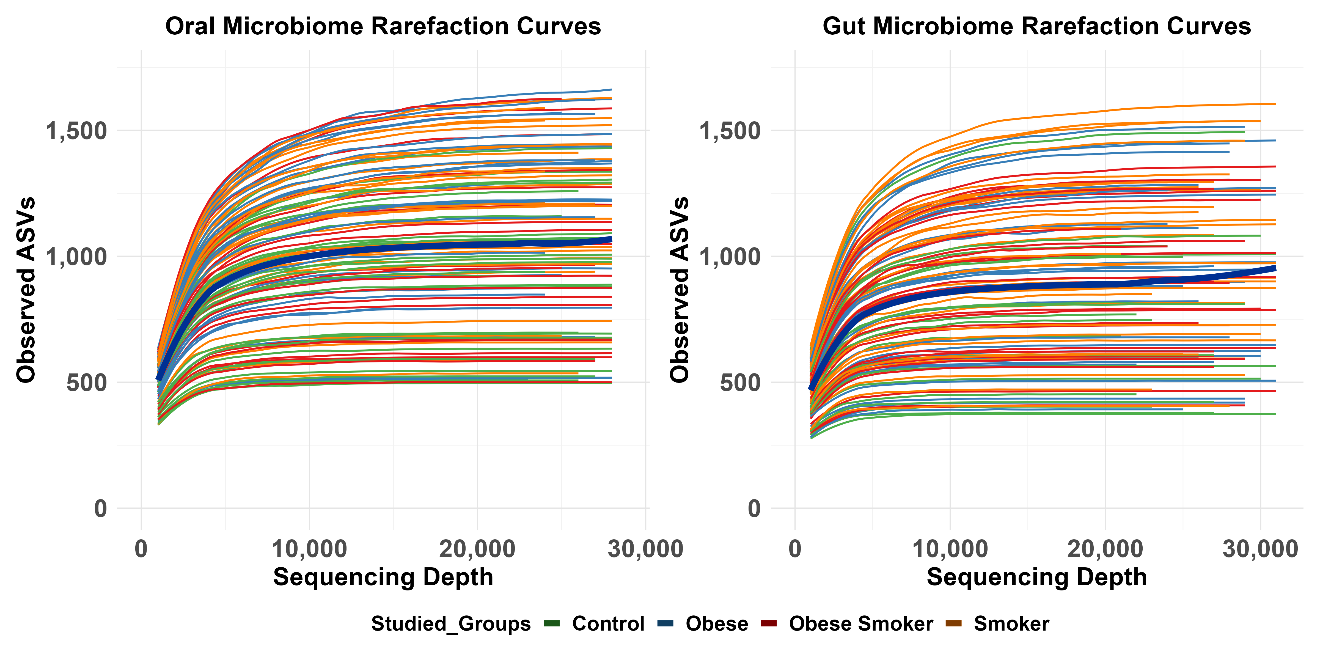
**

**Figure S1.** Variable-Length Rarefaction Curves Showing Microbial Diversity of oral and gut microbiomes.


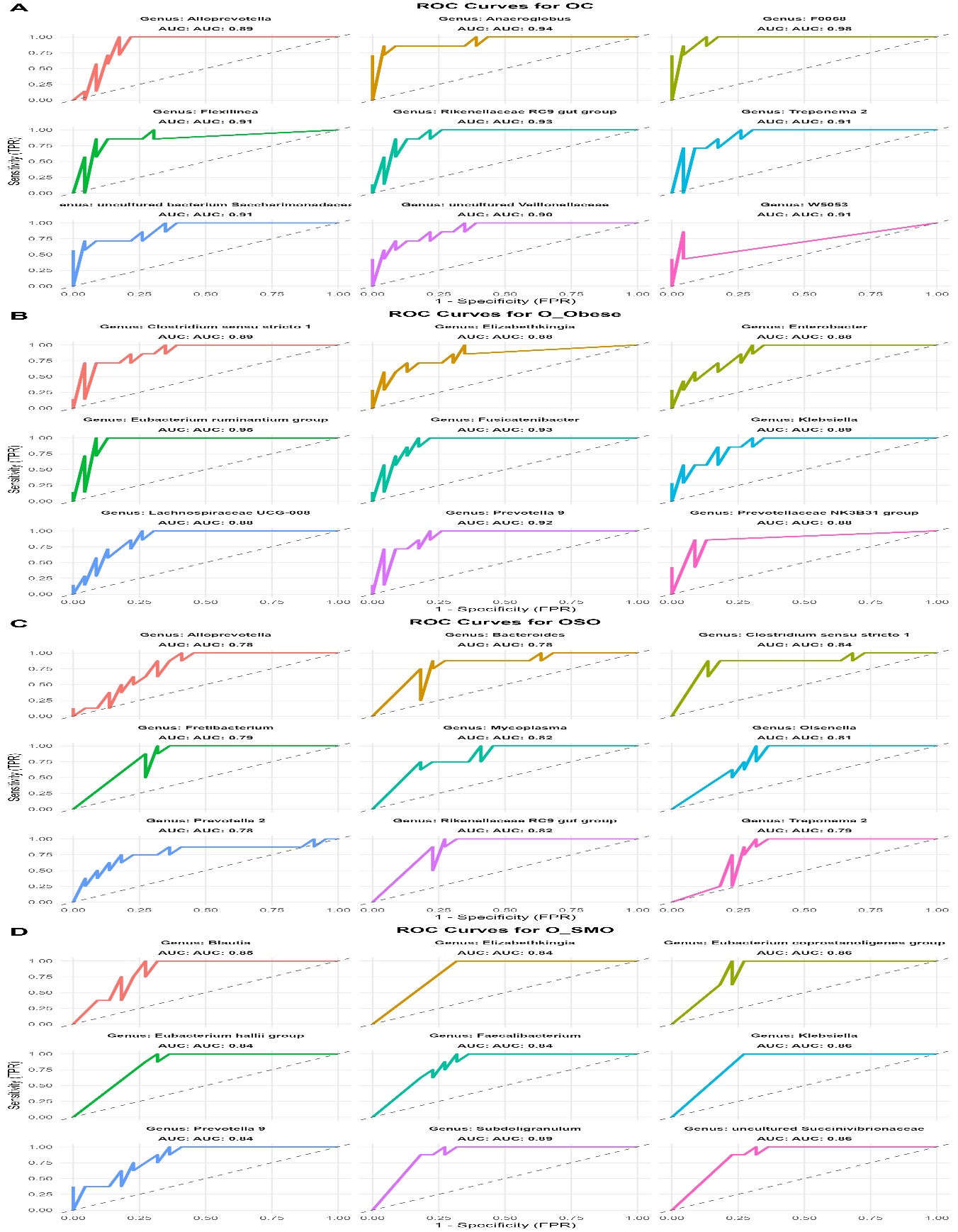


**Figure S2.** ROC Curves for Top Discriminative Bacterial Genera of oral microbiome in the studied Groups.


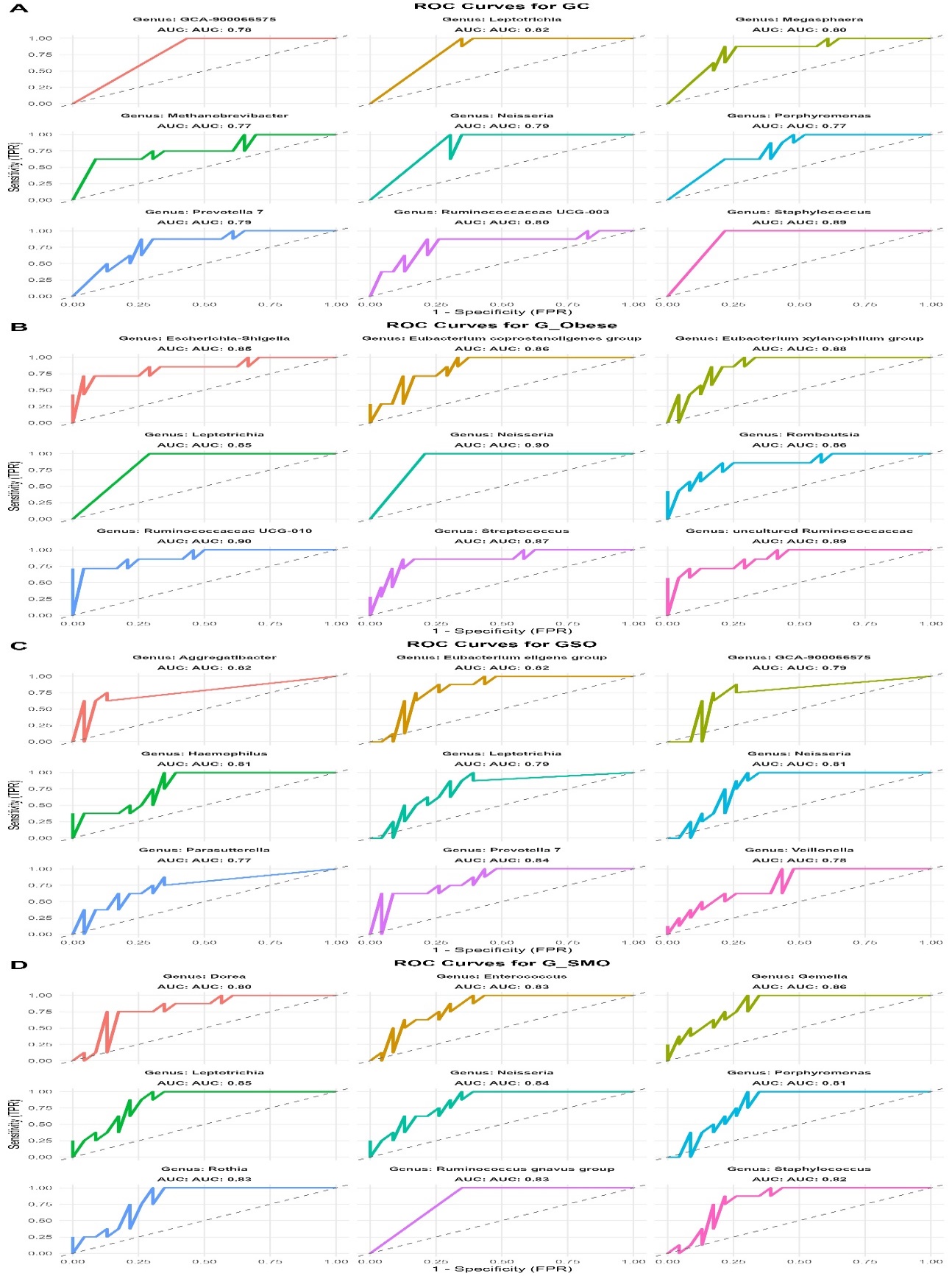
**Figure S3.** ROC Curves for Top Discriminative Bacterial Genera of gut microbiome in the studied Groups

These figures(S2-S3) display Receiver Operating Characteristic (ROC) curves for the most discriminative bacterial genera. The panels show the diagnostic performance for distinguishing the following groups: (A) C = Control individuals, (B) Obese = Obese , (C) SO = Obese-Smokers, and (D) SMO = Smokers. In each subplot, sensitivity is plotted against 1-specificity, and the Area Under the Curve (AUC) values quantify the classification accuracy.


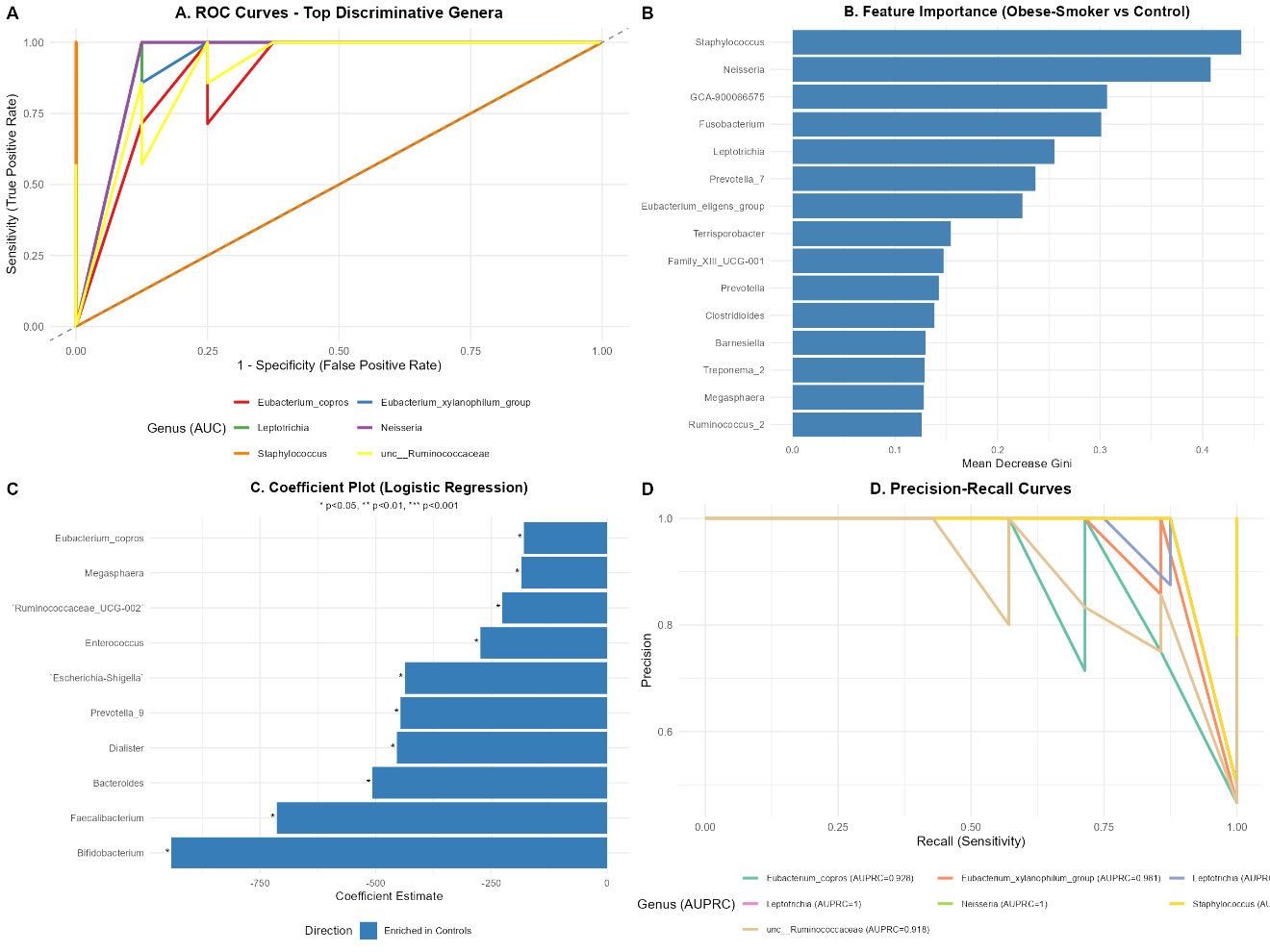


**Figure S4. Machine Learning-Based Analysis of Discriminatory Gut Genera Associated with Obesity and Smoking Status.**

**Multipanel assessment of microbial features distinguishing clinical groups.** Gut genus-level relative abundance data were analyzed to identify taxonomic features capable of discriminating between studied groups.

**(A) ROC curves for top discriminative genera. Receiver** operating characteristic curves showing the predictive performance of individual genera with AUC > 0.75 across the three pairwise comparisons. The diagonal dashed line represents random classification (AUC = 0.5).

**(B) Random forest feature importance. Mean** decrease in Gini coefficient from random forest classification of obese smokers versus controls, indicating the relative contribution of each genus to classification accuracy. Higher values denote greater discriminatory importance.

**(C) Logistic regression coefficient plot.** Direction and magnitude of association for the top 10 genera in the obese smoker versus control comparison. Positive coefficients indicate enrichment in cases (obese smokers); negative coefficients indicate enrichment in controls. Significance levels: *p < 0.05, **p < 0.01, ***p < 0.001; ns, not significant.

**(D) Precision-recall curves.** Precision-recall curves for the top discriminative genera, with area under the precision-recall curve (AUPRC) values in parentheses. Unlike ROC curves, PR curves are particularly informative for imbalanced datasets and emphasize performance on the positive class.


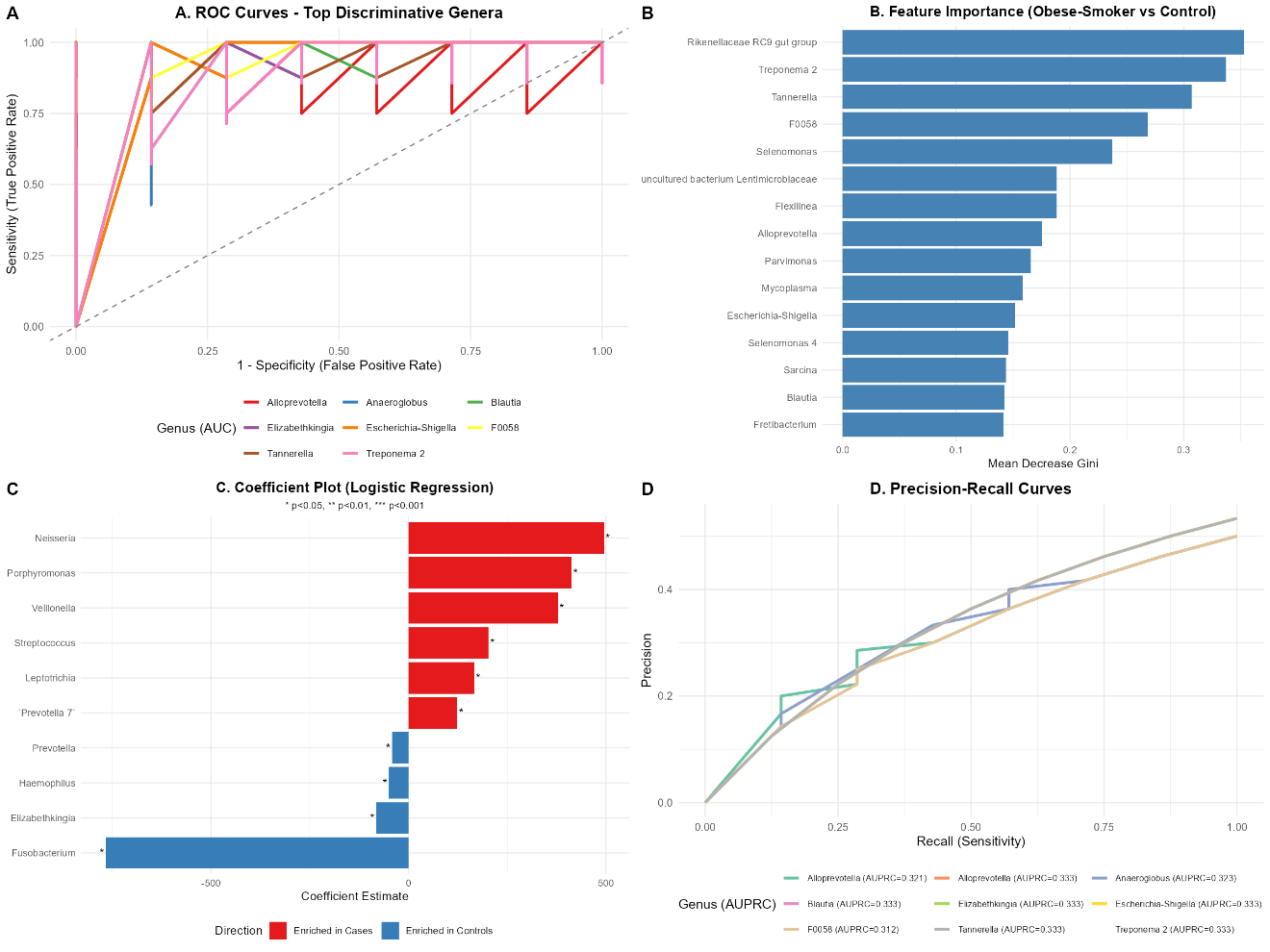


**Figure S5. Machine Learning-Based Analysis of Discriminatory Oral Genera Associated with Obesity and Smoking Status.**

**Multipanel assessment of microbial features distinguishing clinical groups.** Oral genus-level relative abundance data were analyzed to identify taxonomic features capable of discriminating between obese smokers (OSO), obese non-smokers (O_Obese), normal-weight smokers (O_SMO), and normal-weight controls (OC).

**(A) ROC curves for top discriminative genera.** Receiver operating characteristic curves showing the predictive performance of individual genera with AUC > 0.75 across the three pairwise comparisons. The diagonal dashed line represents random classification (AUC = 0.5).

**(B) Random forest feature importance.** Mean decrease in Gini coefficient from random forest classification of obese smokers versus controls, indicating the relative contribution of each genus to classification accuracy. Higher values denote greater discriminatory importance.

**(C) Logistic regression coefficient plot.** Direction and magnitude of association for the top 10 genera in the obese smoker versus control comparison. Positive coefficients indicate enrichment in cases (obese smokers); negative coefficients indicate enrichment in controls. Significance levels: *p < 0.05, **p < 0.01, ***p < 0.001; ns, not significant.

**(D) Precision-recall curves.** Precision-recall curves for the top discriminative genera, with area under the precision-recall curve (AUPRC) values in parentheses. Unlike ROC curves, PR curves are particularly informative for imbalanced datasets and emphasize performance on the positive class.

**
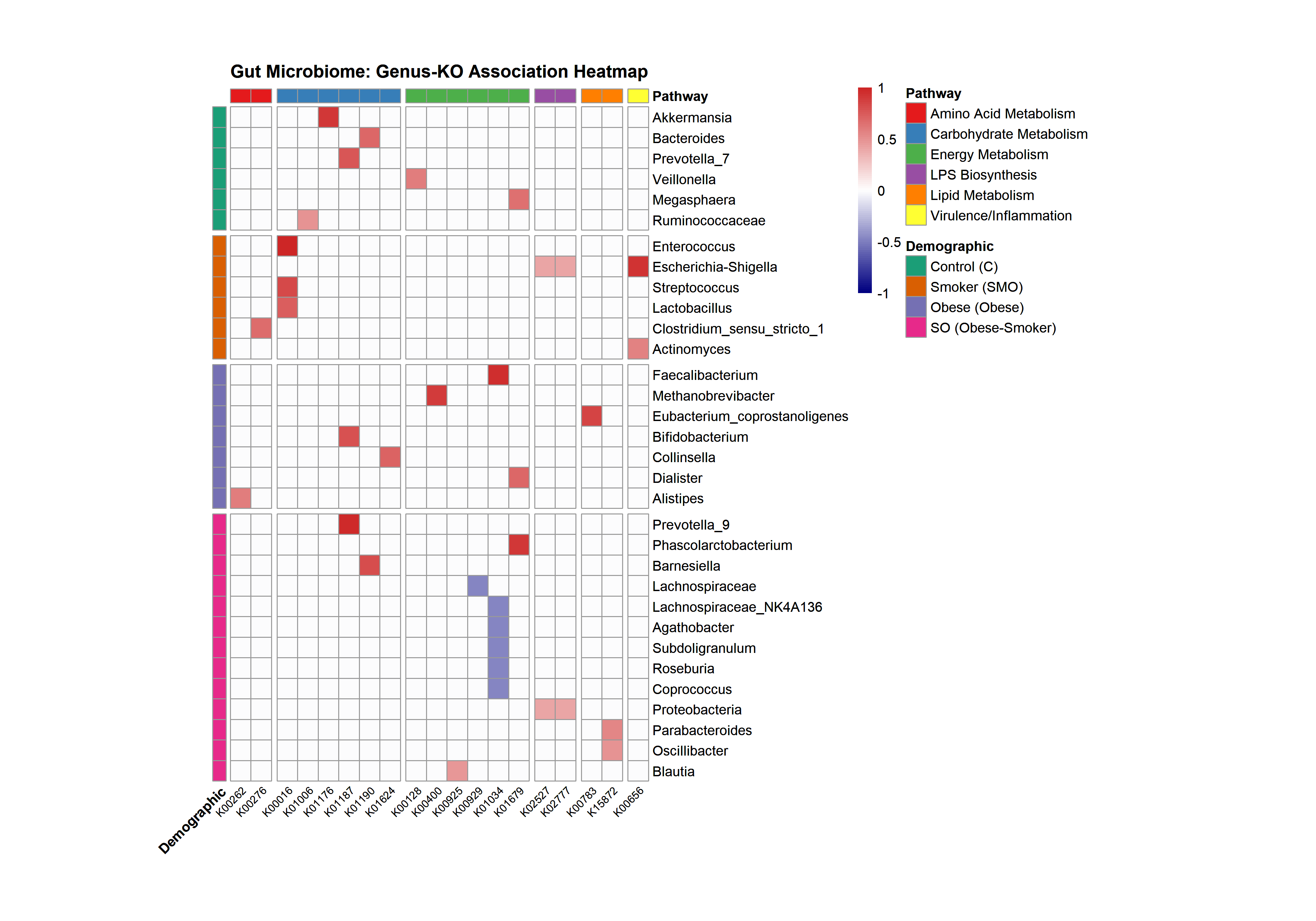
**

**Figure S6**. Gut Microbiome Functional Potential Stratified by Obesity and Smoking **Status.** Heatmap depicting associations between bacterial genera and KEGG Orthologs (KOs) in the gut microbiome of participants. Rows represent bacterial genera grouped by demographic category (Control, Smoker, Obese, and Obese-Smoker). Columns represent KOs grouped by predicted metabolic pathway (2-level). Cell colors represent scaled log2 fold change (log2FC), indicating the strength and direction of association (red, enriched; blue, depleted). Row annotation bars indicate demographic group identity (red, Obese-Smokers; blue, Obese; green, Smokers; purple, Control). Column annotation bars indicate predicted metabolic pathways . Values are scaled to a range of -1 to +1 based on maximum absolute log2FC.


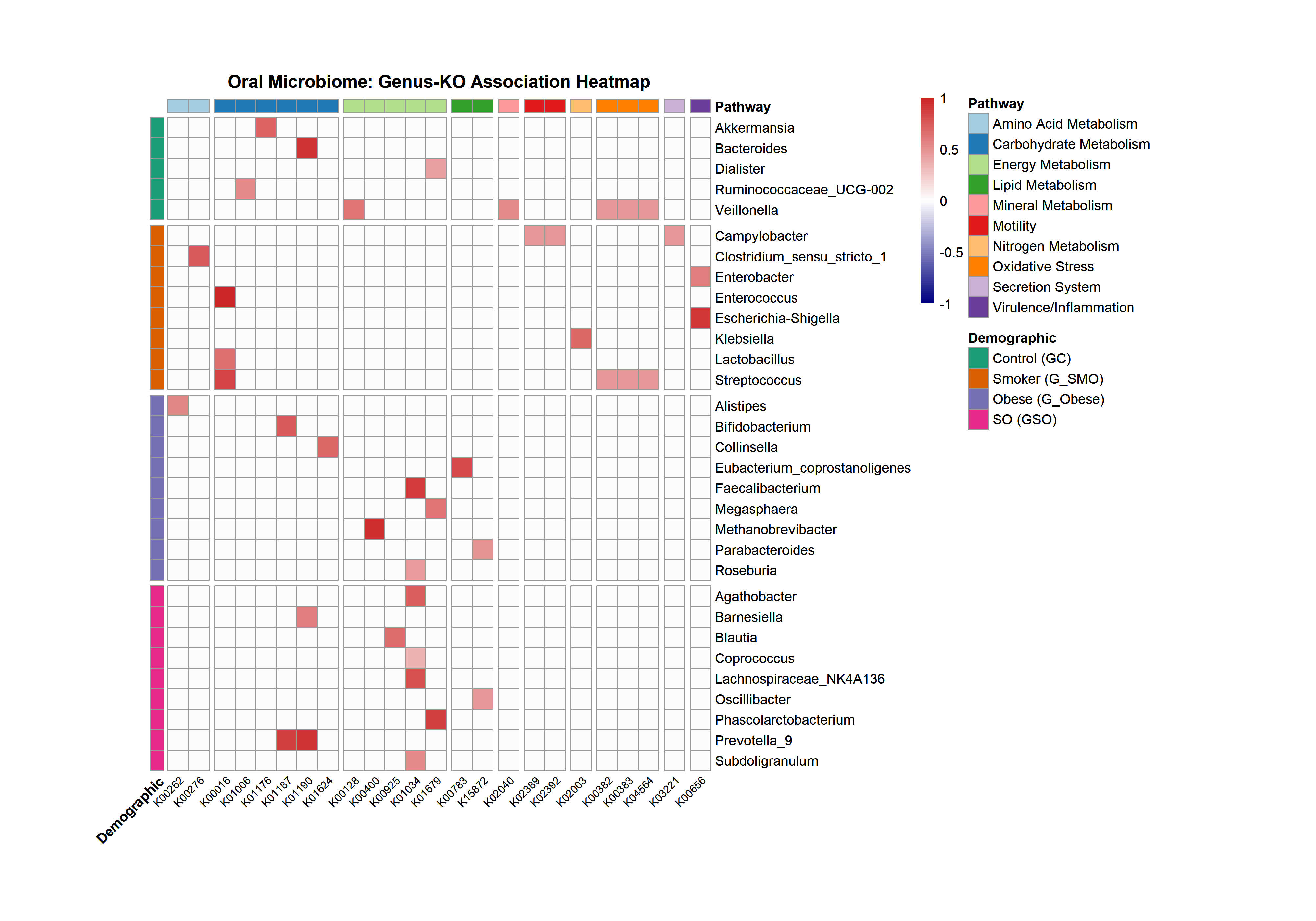


**Figure S7. Oral Microbiome Functional Potential Stratified by Obesity and Smoking Status.** Heatmap depicting associations between bacterial genera and KEGG Orthologs (KOs) in the oral microbiome of participants. Rows represent bacterial genera grouped by demographic category (Control, Smoker, Obese, and Obese-Smoker). Columns represent KOs grouped by predicted metabolic pathway (2-level). Cell colors represent scaled log2 fold change (log2FC), indicating the strength and direction of association (red, enriched; blue, depleted). Row annotation bars indicate demographic group identity (red, Obese-Smokers; blue, Obese; green, Smokers; purple, Control). Column annotation bars indicate predicted metabolic pathways. Values are scaled to a range of -1 to +1 based on the maximum absolute log2 fold change (log_2_FC).
